# Certification or cash prize: the heterogeneous effect of venture competitions

**DOI:** 10.1007/s11187-026-01234-3

**Published:** 2026-06-30

**Authors:** Gaétan de Rassenfosse, Matthias van den Heuvel

**Affiliations:** 1 College of Management of Technology, Ecole polytechnique fédérale de Lausanne, Lausanne, Switzerland; 2 The Brattle Group, Paris, France

**Keywords:** Cash prize effect, Certification effect, Early-stage financing, Expert evaluation, Startup survival, Venture competition, D22, D80, G24, L26, M13, O31

## Abstract

Venture competitions usually reward winners with a certification of their startup’s quality and a cash prize. We model and estimate the impact of these rewards on startups’ performances using original data on about 1000 startups that have participated in the leading Swiss venture competition. We find that winning in the competition improves startups’ business success on average. However, it does not affect all startups equally. Certification has a long-term positive effect on startups whose quality can be reliably assessed by the competition’s judges. The cash prize, however, only has a short-term impact on startups with low running costs.

## Abstract (plain language summary)

Badges beat bags of cash. Studying nearly a thousand startups in a leading national venture competition in Switzerland, we find that an expert endorsement (a public win that signals quality) has a lasting effect on startup survival and fundraising—especially in medtech/biotech and other science fields. Cash awards, in contrast, extend the runway mainly for low-cost digital ventures and do not raise investor funding; the boost fades after 2 to 3 years. The implication for program design is clear: strengthen the information value of competitions by investing in credible expert evaluation. For time-constrained entrepreneurs, choose your competition wisely: cash burns off, but the credibility of the jury for your specific venture goes a long way to making the quality signals stick.

## Introduction

Venture competitions, events in which startups compete to receive money, mentoring or recognition, have become a key institution in the startup ecosystem. According to Howell (2020), about 15% of US startups that secured their first seed or series A financing between 2009 and 2016 have won such a competition. Changes in the investment strategy of venture capitalists (VCs) are reportedly behind the rise of venture competitions. Faced with falling costs of starting a venture, investors have increasingly adopted a “spray and pray” investment approach (Ewens et al., 2018). VCs are said to provide more limited funding and governance to a greater number of startups—that they are then more likely to abandon.

In this changing environment, venture competitions can provide welcome support to early-stage startups. First, they usually supply a cash prize to the winning startups at a stage where acquiring financing is challenging, thereby mitigating potential financial constraints. Second, they often offer some form of mentoring or networking, presenting fledgling entrepreneurs with opportunities to improve their business. Finally, winning a venture competition can act as a certification of quality, providing valuable information about a startup’s probability of success to both founders and investors.

Research on venture competitions is still in its infancy. There is no consensus among scholars as to precisely why these competitions affect startups’ business success. McKenzie (2017) finds that it is the cash prize effect that matters most, whereas Howell (2020) finds that venture competition’s main benefit is the certification effect. Smith and Viceisza (2018) argue that it is the financing that ultimately matters, and not the signal of quality that winning a competition provides. The present paper aims to reconcile these conflicting results by exploring factors that drive the certification and cash prize effects.

We start by developing a theoretical model of venture competitions’ impact on participating startups. Our model yields the following testable predictions. First, certification has a long-lasting effect and is particularly useful to startups in sectors that experts are adept at screening. Second, because the cash prize’s main impact is to extend startups’ runway, it has a stronger impact on startups with relatively low running costs, and its effect is limited in time.

To test these predictions, we have obtained original and detailed data on 987 startups that participated in Venture Kick, the leading Swiss venture competition, in the period 2008–2017. Switzerland has a vibrant entrepreneurial scene, ranking fourth in the OECD in terms of the amount of venture capital investments per capita. The competition is organized in three eliminatory stages and provides up to $130,000 to winning startups. This set-up is ideal for our purposes as we exploit the multi-stage nature of the competition to tease out the certification and the cash prize effects. Because winning the first stage is very often a startup’s first certification of quality but comes with barely any financing, we argue that it primarily captures the certification effect. By contrast, winning the last stage comes with a large cash prize and, therefore, predominantly captures the cash prize effect. Our main identification strategy exploits detailed data on grades given by the judges, which we use to control for differences in startup quality.

Regarding the certification effect, we find that, on average, winning the first stage of the competition increases survival rates 2 years after the competition by 22 percentage points. It also increases the probability of securing external financing at any point after the competition by 14 percentage points. This effect is a significant one considering that the unconditional probability of securing funding after the competition is 26% in our sample. However, not all startups benefit equally from the certification. In line with our model’s first prediction, we find that the certification effect is particularly strong among startups whose quality can be more objectively assessed by the competition’s judges, notably science-based startups (à la Scott et al., 2020). Winning the first stage increases their survival rate 2 years after the competition by 41 percentage points. This effect is long-lasting, raising survival rates by 35 percentage points 6 years after the competition. By contrast, startups whose quality is harder to assess, such as internet, mobile, and software (ICT) startups, do not seem to benefit from the certification. For these startups, the certification is too noisy a signal of quality.

Turning to the cash prize, we find that, on average, winning the third stage has no significant effect on participating startups’ future survival rates or funding outcomes. Once again, though, this result hides heterogeneity across sectors. Indeed, ICT startups with lower running costs are better able to take advantage of the cash prize than the more capital-intensive science-based startups. As a result, receiving the $100,000 prize significantly extends the runway of ICT startups, but has no impact on science-based startups. We find that the cash prize improves the survival rates of ICT startups by 23 percentage points in the first 2 to 3 years after the competition, the effect vanishing once the money is exhausted. Finally, the cash prize never seems to affect startups’ ability to attract external investors, a sign that the prize money does not fundamentally alter a startup’s prospects.

Our results are robust to alternative specifications and identification strategies. Most notably, we implement a sharp regression discontinuity design (RDD) that exploits the fact that startups ranked just above the cutoff receive treatment while those below do not. We also use propensity score matching and errors-in-variables models that allow grades to be observed with some level of imprecision. All approaches point in the same direction.

Another notable finding is that the certification effect seems to work in two ways. First, it acts as a type-revelation mechanism. The feedback provides both winning and losing entrepreneurs with information on their startup’s quality that can help them adjust their probability of success. Like Yu (2019) and Howell (2021), we find evidence that losing startups shut down earlier than they would have otherwise. Second, when the judges’ evaluation of startups’ quality is expected to be more reliable, as in the case of science-based startups, certification provides valuable information on the startups to investors. As a result, winning startups improve their chances of securing external financing after the competition.

Regarding generalizability, our findings only apply to startups that would participate in a venture competition like Venture Kick. We cannot formally test the representativeness of our sample given the lack of comprehensive, country-wide data on Swiss startups. However, Venture Kick’s participants exhibit a value distribution similar to the broader startup population, with a handful of global industry leaders such as Climeworks or Mindmaze and a host of flawed, low-quality ventures. Moreover, participants in Venture Kick have rates of exit and post-competition funding similar to the startups in Howell (2020)’s US venture competitions. Beyond sample representativeness, our cross-sectional finding is itself context-specific: in competitions with different judging panels, the ICT-versus-science pattern may not replicate. We see this as an open question for future work.

The paper makes three contributions to the nascent literature on venture competitions. First, it offers a different view on when certification matters. Howell (2020) finds that the certification effect is largest for Internet- and software-based ventures and attributes it to a demand-side mechanism: low-cost ventures yield more marginal entrants, and a positive signal helps investors experiment with high-risk early bets. We document the opposite cross-sectional pattern: in Venture Kick, ICT startups receive no certification benefit while science-based startups do. We propose a complementary, supply-side mechanism: judges produce informative signals only in fields where they have the expertise to assess quality. Both mechanisms can operate, and which dominates is an empirical question for future work. Second, we reconcile McKenzie (2017) and Howell (2020) on cash prizes by arguing that the cash effect operates through runway extension, and, therefore, depends on a venture’s running cost relative to the prize, rather than on the absolute prize amount. Third, we exploit a multi-stage competition in a single institutional setting to disentangle certification and cash effects within one design, complementing prior work that studies each mechanism in a different competition. More broadly, our findings add evidence in a structurally different context to a literature that remains thin and where existing findings disagree.

The paper is organized as follows. The next section provides background information on venture competitions. Section 3 presents our model and its theoretical predictions. Section 4 presents Venture Kick, discusses the data, and provides descriptive statistics. Section 5 details our preferred empirical strategy. Section 6 tests the predictions of our model, investigating Venture Kick’s average and industry-specific impact. Section 7 confirms the robustness of our results, and Sect. 8 concludes.

## Background

The past two decades have seen a sharp decrease in the cost of starting a new high-technology startup or experimenting with new ideas.^1^ This decrease in costs has sparked change both in the way venture capitalists (VCs) invest and the type of investors that are now able to provide support to early-stage ventures (Ewens et al., 2018; Nanda & Rhodes-Kropf, 2016). VCs have increasingly adopted a “spray and pray” investment approach (Ewens et al., 2018), whereby they make a large number of small investments in early-stage ventures with limited due diligence and governance in the hope that a handful of these ventures will hit a home run. This reduction in VC firms’ governance and the fall in the cost of starting a business help explain the emergence of new early-stage financial intermediaries such as venture competitions, accelerators/incubators, angel networks, or crowdfunding platforms that provide scalable, lower-cost forms of mentorship (Ewens et al., 2018).

In the past decade, a literature has emerged to study these new, and increasingly important, financial intermediaries including accelerators, incubators, and university entrepreneurial programs (Chan et al., 2020; Contigiani & Young-Hyman, 2022; Gonzalez-Uribe & Leatherbee, 2017; Yu, 2019). Angel investors have also been studied in recent years with Kerr et al. (2011) and Lerner et al. (2018), showing that the participation of angel investors in early-stage ventures increases their survival, employment, and follow-on financing. The authors including Mollick (2014); Hildebrand et al. (2017) and Gafni et al. (2019) analyze the crowdfunding phenomenon spearheaded by platforms like Kickstarter. Finally, venture debt lenders, specialized institutions that provide loans to high-growth startups, have been studied by de Rassenfosse & Fischer (2016) and Hochberg et al. (2018), among others.

Venture competitions have so far received less academic attention, with the notable exceptions of McKenzie (2017) and Howell (2020). Yet, they are an important actor in the startup ecosystem. According to Howell (2020), 14.5% of the startups reported on the American data platform CB Insights that secured their first seed or series A financing between 2009 and 2016 won a venture competition. These competitions are events in which a number of startups compete through one or more stages. Their founders pitch their technologies and business models to a panel of judges in the hope of receiving funding, mentoring, or certification of quality. The literature has found that venture competitions benefit startups because of either their certification or their cash prize.^2^

Through their certification, the competition’s judges provide useful information about the value and potential of participating startups (Scott et al., 2020). This role is essential because information acquisition is crucial in the investment decision of early-stage investors (Bernstein et al., 2017). Gompers et al. (2020) find that selecting the right startup is the most important determinant of VCs’ success. Moreover, information asymmetry between investors and entrepreneurs has long been identified as a problem that hinders investment in startups (Amit et al., 1990), and this problem might have become even more acute today. Indeed, the fall in costs has lowered the barriers to entry, making it harder for VCs and other early-stage investors to determine each startup’s quality (Ewens et al., 2018). Venture competitions’ certification of quality may, therefore, reduce the information asymmetry problem. These competitions also inform the entrepreneurs about their startup’s probability of success, guiding their decision to proceed or stop further development efforts. Åstebro and Gerchak (2001) find that the advice to proceed or not with a venture provided by Canada’s Inventor’s Assessment Program generated higher benefits for society than the costs of running the program. More specifically, in the context of venture competitions, Howell (2020) puts forth evidence of a significant certification effect. The author finds that winning a competition seems to primarily serve a certification function, signaling quality to the market and reducing search frictions between VCs and entrepreneurs. Yu (2019) and Howell (2021) find that accelerators and venture competitions provide informative feedback to founders. They argue that this feedback resolves uncertainty around company quality sooner, which accelerates the shutdown of losing ventures.

Turning now to the cash prizes, venture competitions have the potential to alleviate a startup’s financial constraints. A wealth of evidence suggests that liquidity constraints affect the viability of startups, starting with Holtz-Eakin et al. (1994), and that the growth of most firms is constrained by internal finance (Carpenter & Petersen, 2002). Moreover, Clementi and Hopenhayn (2006) have shown that borrowing constraints have important implications for firm growth and survival. By providing a cash prize, venture competitions can typically alleviate these financial constraints. McKenzie (2017) provides empirical evidence of this effect. Studying a competition in Nigeria that awarded almost $50,000 to its winners, the author finds that winning is helpful mainly because of the large cash prize. Looking at the Shark Tank competition, Smith and Viceisza (2018) also conclude that it is the financing that ultimately matters, and not the signal of quality that winning the show provides.

In order to reconcile these different results, we propose that variations in the competitions’ set-ups need to be considered. Everything else equal, it seems logical that a cash prize will be relatively more useful in Nigeria (McKenzie, 2017) than in the USA (Howell, 2020), where startups have access to numerous alternative sources of financing. Moreover, the size of the cash prize relative to the cost of living certainly matters. In the Shark Tank’s case, the financing provided is larger than in typical venture competitions, which may help explain why the authors observe a financing effect (Smith & Viceisza, 2018).^3^ Overall, these different results suggest that not all participants benefit equally from venture competitions (Lyons & Zhang, 2018). They call for a more fine-grained understanding of the factors that affect venture competitions’ effects on startup performance.

## Theoretical predictions

We propose a theoretical model that considers circumstances under which the certification and the cash prize effects will matter to different degrees. We consider two types of agents and one institution that operate with imperfect information. Entrepreneurs, who do not know the exact quality of their startup, have to decide each period whether they should continue their activity. Investors have to decide whether to invest in the entrepreneurs’ startups based on their prediction of the venture’s quality. Finally, a venture competition provides a certification of quality and a cash prize to participants. The certification of quality provides information that allows both entrepreneurs and investors to better predict startup quality. The cash prize helps startups cover their operating costs, which vary by technology. We leave the full details of the model to Appendix 1. The present section focuses on the testable predictions we can draw from the model and provides some key intuitions for them.

The first set of propositions relates to the certification effect offered by the competition. In our data and in the literature, there is evidence that the ability of judges to screen startups varies across technology types. Consequently, we argue that investors and entrepreneurs are more responsive to certification in sectors in which technologies can be objectively assessed. The information that the certification provides permanently shifts the agents’ expectations of the startups’ future profitability. We therefore expect the certification effect to be long-lasting, only losing some of its potency over time as agents receive new information about startups’ true quality.

- **Proposition 1: ***Certification is particularly useful to startups in sectors that experts are adept at screening.*
- **Proposition 1.a: ***The certification effect is persistent over time.*

The second set of propositions relates to the cash prize effect that comes with winning the competition. We argue that the main role of the cash prize is to extend startups’ runway. It does not, however, directly impact startup quality or its estimation. As a result, the magnitude of the cash prize effect hinges on how much extra runway it affords. Furthermore, as the cash prize does not affect the intrinsic quality of the startup, we expect its effect to be short-lived.

- **Proposition 2: ***The cash prize has a stronger impact on startups with relatively low running costs.*
- **Proposition 2.a: ***The cash prize effect is mostly temporary.*

## Data

Switzerland and Venture Kick, its leading venture competition, offer an ideal setting to test the predictions of our model. Indeed, Switzerland hosts a thriving entrepreneurial environment with many active early-stage investors. According to Dealroom, Switzerland is the fourth country worldwide with the highest amount of venture capital investments per capita (after Singapore, the USA, and Israel).^4^ This setup is essential because it means that certification will matter, and the cash prize will not be the only available source of funding. Additionally, the venture competition we study needs to provide both certification and a cash prize in a way that allows us to estimate their impact separately. These rewards also need to have been provided in a consistent manner throughout the years. Venture Kick satisfies all these criteria. It has maintained a very similar reward structure in terms of certification/publicity and cash prize during the entire study period. Moreover, the judges have graded startups using the exact same guidelines and criteria since the start of the competition in 2007. Finally, because Venture Kick is open to all technology types, we are able to observe significant variability in the parameters that matter in our model: running costs and experts’ ability to predict the startup’s future success.

### The Venture Kick competition

Venture Kick is a Swiss venture competition based in Lausanne and Zurich. It has awarded more than $59 million to 1024 startups since its launch in 2007 (Venture Kick, 2023). It aims to increase the number of spin-offs at Swiss universities, accelerate the time-to-market, and raise the attractiveness of these young companies to professional investors. Venture Kick has become an important actor in the Swiss startup scene. Using data from CrunchBase, we find that 22% of all the startups based in Switzerland founded between 2006 and 2019 that completed a round of series A financing received money from Venture Kick. More importantly, seven of the 13 best-ranked startups in this group, including MindMaze, Switzerland’s first unicorn at $1+ billion valuation, are Venture Kick alumni.^5^ Venture Kick has, therefore, supported a disproportionate share of Switzerland’s most successful startups. This record of identifying successful startups supports the argument that Venture Kick has the potential to be a credible and well-regarded source of certification.

The competition focuses on very early-stage ventures. To be eligible to participate, companies should not yet be incorporated, or only very recently, and cannot have received funding from professional investors. These requirements imply that Venture Kick is usually involved in startups’ development before any other external investors or entrepreneurship programs. Additionally, participating startups should have links with a Swiss academic institution or research center and plan to establish their company and create jobs in Switzerland. Conditions attached to the grant money have not changed significantly during the years of our study, from 2007 to 2017.^6^ The first $10,000 and $20,000 are given as a grant with a moral obligation to repay in case the startup becomes successful. The final $100,000 was initially also given as a grant, but this feature changed in 2013, when Venture Kick started taking a 1 to 3 percent share in the winning companies.

Venture Kick is structured as a three-stage, 9-month, private competition open several times a year to a new group of eight startups. Not all startups that apply are accepted; around 20% of applicants are rejected outright.^7^ The competition’s judges are drawn from a pool of more than 150 private and institutional investors, startup experts, and industry representatives. Twenty-seven percent of the judges list Internet and Mobile and Software as their main area of expertise while the rest are experts in more science-based technologies (e.g., biotech and medtech).^8^ These judges register for the competitions based on their availability without knowing the participants.

In the first stage (stage 1), startups pitch their business idea in front of a jury composed of an average of nine judges. Each jury member grades the startups on eleven different criteria and votes for the startups they deem worthy of advancing to the next stage.^9^ These four winning startups receive $10,000, and Venture Kick promotes them on its website and social media. Winning startups also gain the right to display the competition’s logo on their own website—a logo that is identical whether the startup wins only stage 1 or all three stages.^10^ Finally, they secure access to the first two, out of three, kicker camps. These camps are 2-day events where entrepreneurs receive mentoring and are put in contact with accomplished entrepreneurs and industry experts.

In the second stage (stage 2), 3 months later, the startups have to present their business case in a similar setting to a different set of judges. Two startups are selected for the third, and final, stage (stage 3). These two startups receive $20,000 as well as the same written publicity as in stage 1. They also gain access to the last kicker camp. Six months later, in stage 3, the last two startups compete to win the competition and the $100,000 prize attached to it. Once more, Venture Kick communicates about the ultimate winner’s success. Figure 8 in Appendix 4 illustrates the whole process. In addition, after each stage, Venture Kick provides successful and unsuccessful participants with private feedback on their performance. This feedback includes a video of their presentation and subsequent Q&A, their grades, and a report that contains all the jury’s comments and open questions about their project’s viability.

### Data provided by Venture Kick

Venture Kick has provided us with anonymized data on its competition and on 987 startups that have participated in a total of 305 rounds of competition (spread across stages 1, 2, and 3) over the years 2008 to 2017. The data include information on the startups (e.g., location, link with university, industry, gender of founder) and their performance during and after the competition.^11^ These data form one of the strengths of our paper as all the startups have gone through exactly the same process, with the same set of rules, and were motivated by the same financial and non-financial incentives. Also, the relatively low number of participants per competition (a maximum of eight) means that the judges have the time (around 30 min) to scrutinize each startup adequately. As this process is repeated up to three times, each startup that competes in stage 3 will have spent 90 min with Venture Kick’s judges. This setup contrasts with previous studies. Howell (2020)’s larger sample includes 87 different competitions that each had between six to 275 participants and one to 178 judges. In McKenzie (2017), there is only one paper-based competition during which the 6000 applications are assessed by an algorithm and an evaluator.^12^ We exploit the consistency and quality of the grading data in our identification strategy, as explained in Sect. 5.

Venture Kick has recorded every grade and vote given by the jury members in all three stages since 2008 and has shared them with us. In stage 1, each judge grades the startups on ten elements summarized by a “People” and a “Project” grade and an eleventh grade denominated “Gut Feeling.” In stages 2 and 3, the “Gut Feeling” grade remains, but the other ten grades are replaced by ten new grades summarized by a “People & Project” grade and an “Achievements & Development” grade. Grades are given on an absolute scale from 0 (worst) to 6 (best). This absolute scale allows us to identify weaker cohorts, providing some useful variation in the quality of both losers and winners. Because the variance in grades given to different startups decreases through the stages as the quality of the participants becomes consistently higher, we standardize each grade at the stage level. For our analysis, we decided to aggregate our eleven grade variables into one single measure by using a principal component analysis (PCA).^13^ Table 8 in Appendix 5 shows the eigenvalues of the PCA on all the grades given in stage 1. Using the Kaiser Criterion, dropping all components with eigenvalues below 1, we retain only the first principal component that accounts for 76% of the variance in grades. We interpret this first component as being a measure of startup quality. Moreover, Table 9 shows that the first component loads similarly onto all the grades, which is what we would expect as startup quality should be reflected in all the grades. Henceforth, the grade variable we use in the analysis is the measure produced by the PCA, unless specified otherwise. On the startups themselves, we have data on their characteristics, including industry, date of creation, gender of the main founder, and the university it is affiliated with.

### Additional data collected

Testing the model’s predictions requires data on both the decisions of the entrepreneurs and the investors. To capture the entrepreneur’s decision to pursue their activity, we use data on startups’ survival status in the years after the competition. To capture the investor’s decision to invest in a given startup, we use data on whether and how much startups are able to secure external funding in the years after the competition. The data collection process was performed under our close supervision by Venture Kick to preserve the anonymity of the startups’ information.

To construct the annual survival variable, we manually verified both whether and when each of the 759 startups that competed between 2008 and 2015 stopped their activity during the period up to the 1st of January 2018.^14^ To do so, we relied on the following sources: Cantonal registries of commerce; the startup’s website status and activity—if the website was down we used the Internet Archive’s Wayback Machine^15^ to find out when it stopped being active; LinkedIn’s staff profile and startup pages; Facebook and Twitter activity; CrunchBase’s databases and other resources like newspaper articles and Venture Kick’s own resources. We followed the same process to obtain data on startup exit (IPO & Acquisitions).^16^ To build our funding variables, we manually collected funding information on CrunchBase for the 970 startups that competed between 2008 and 2017 for the period up until the 1st of January 2020.^17^ These data provide the number of funding rounds, their dates, and the amount of funding received for all participating startups. We also used patent data from the PATSTAT database to measure the startups’ innovation output and matched 693 patents to 104 startups in our sample.

Note that having access to annual outcome data is quite rare in the literature. These data will allow us to differentiate between the short-term and long-term effects of the certification and cash prize.

### Descriptive statistics

Table 1 shows descriptive statistics concerning the startups in our sample, depending on how far they progressed into the competition. This table yields interesting facts about participants and winners. First, software and internet and mobile startups do not perform well in the competition. Although they represent 43% of the sample, only 17% of the winners of stage 3 come from these two sectors. At the other end of the spectrum, medtech, biotech, and electronics and mechanics startups are over-represented among the winners of stage 3 (58 percent). Second, two universities dominate the competition. The two federal institutes of technology in Lausanne (EPFL) and Zurich (ETHZ) represent 44% of the participants but 72% of the winners. Third, women entrepreneurs are under-represented, with 89% of the startups having declared a man as the main founder. However, they exhibit roughly the same success rate as men.

As could be expected, startups that go further in the competition tend to have a higher survival probability. Twenty-seven percent of the startups that lost in stage 1 are alive 5 years after the competition versus 93% for startups that won the final stage (last column). They also tend to secure more financing, have more employees and larger turnover, and obtain more patents in the years following the competition. Overall, the data tell us that winning the competition correlates with better performances after the competition.

Additionally, Table 1 shows that startups participating in Venture Kick are very similar to their peers in other developed countries. Indeed, in the USA, Howell (2020) finds that 3% of the startups that participated in the studied venture competitions have exited, which is the exact same number as in our sample. Moreover, 24% of these American startups secured funding after their competition compared to 26% after Venture Kick. The similarities between the startups in our sample and in similar studies bolster the external validity of our findings.

We now turn to our empirical strategy to explore the effects of venture competitions.

**Table 1:** Descriptive statistics: characteristics and outcome

	Full sample	Lost in stage 1	Lost in stage 2	Lost in stage 3	Winners
	Mean	Mean	Mean	Mean	Mean
*Categories:*					
Software	0.24	0.31	0.26	0.16	0.08
Internet and mobile	0.19	0.20	0.20	0.18	0.09
Medtech	0.12	0.07	0.13	0.17	0.23
Electronics and mechanics	0.10	0.05	0.11	0.14	0.19
Biotech	0.09	0.04	0.11	0.15	0.16
Cleantech	0.07	0.04	0.09	0.10	0.09
Materials and chemicals	0.03	0.03	0.04	0.03	0.02
Micro-nano technology	0.03	0.01	0.02	0.06	0.07
Others	0.05	0.09	0.04	0.02	0.02
*Universities:*					
EPFL	0.21	0.13	0.22	0.31	0.33
ETH	0.23	0.17	0.25	0.26	0.39
*Miscellaneous:*					
Main founder is a man	0.89	0.86	0.94	0.88	0.88
Average grade in stage 1	3.85	3.32	4.22	4.34	4.49
Average grade in stage 2	3.97	.	3.56	4.25	4.40
Average grade in stage 3	4.27	.	.	3.89	4.62
*Outcomes:*					
Alive after 2 years	0.61	0.36	0.70	0.84	1.00
Alive after 5 years	0.52	0.27	0.59	0.73	0.93
Received funding after 2 years	0.19	0.06	0.19	0.30	0.46
Received funding after 5 years	0.22	0.07	0.19	0.35	0.62
No. of funding rounds after 2 years	0.40	0.20	0.25	0.53	0.72
No. of funding rounds after 5 years	0.81	0.48	0.51	0.93	1.40
No. of patents after 2 years	0.22	0.04	0.15	0.28	0.51
No. of patents after 5 years	1.74	0.15	1.49	2.42	3.05
No. of employees after 2 years	7.06	.	5.24	7.52	9.64
No. of employees after 5 years	14.70	.	11.39	13.71	20.63
Turnover after 5 years, kCHF	810.85	.	351.12	656.98	1623.60
Acquired/IPO’ed	0.03	0.01	0.04	0.09	0.05
Observations	983	433	267	129	121

*Note:* This presents descriptive statistics for the full sample and conditional on their results in the competition. A number of rounds, patents, employees, and turnover are reported for the sub-sample of startups that are still alive. The total number of observations for the first eleven outcome variables is 713, 428, 945, 606, 432, 224, 468, 234, 331, 176, and 171, respectively. Data on employees and turnover were provided by Venture Kick and obtained through a voluntary annual survey; not all ventures responded. Furthermore, the data are collected only for ventures that reached at least stage 1

## Empirical strategy

Our main empirical challenge is to separately estimate the certification and cash prize effects. We exploit differences across the competition’s stages to disentangle the two: winning stage 1 primarily shifts the certification signal, whereas winning stage 3 mostly captures the cash prize effect. We then outline our identification strategy, which leverages detailed judges’ grades.

### Disentangling certification and cash prize effects

We test for the presence of the certification effect using stage 1 because it is mainly through participation in this first stage that entrepreneurs and investors obtain information about a startup’s quality. Indeed, as Venture Kick is intended for very early-stage startups that professional investors have not yet scrutinized, stage 1 plays a crucial certifying role. Participating in stage 1 provides startups with feedback that is very often the first expert assessment of their potential, encouraging winners to pursue their ventures while discouraging losers. In addition, winning stage 1 usually provides startups with the first credible and informative signal of their quality for outside investors. This signal takes the form of an award and some online publicity—usually the first public endorsement that these startups receive.^18^ It also comes with increased online visibility—97% of the startups that won stage 1 have a profile on CrunchBase compared to only 23% of the losers. Furthermore, stage 1’s $10,000 cash prize is not consequential. To put this number in perspective, the median monthly salary in Switzerland stood at around $6500 in 2019.

Winning stage 2 is mainly about reinforcing the certification and learning effect of Venture Kick. Upon winning stage 2, startups receive $20,000 and some more publicity. The money remains limited, and the publicity is somewhat redundant, reinforcing the certification effect.^19^

We use stage 3 to test for the cash prize effect: winning stage 3 yields $100,000—more than three times the combined prizes of stages 1 and 2—raising the total funding received to $130,000. The certification effect plays a more limited role at this stage. The comparison underlying the stage 3 estimate is between a stage-3 winner and a stage-3 loser, both of whom have already won stages 1 and 2 (and are thus of high quality), both of whom appear on Crunchbase, and both of whom have already been the subject of Venture Kick articles, tweets, and other publicity. The marginal third-party endorsement from winning stage 3 comes on top of two prior public wins and is, therefore, much smaller than the marginal endorsement from winning stage 1, where the comparison is between visibility and invisibility (97% of stage-1 winners have a Crunchbase profile, versus only 23% of losers). The stage-3 estimate thus predominantly captures the marginal effect of the $100,000 cash, with a small residual marginal-certification component.

Each stage provides *some* certification and *some* cash, so we cannot cleanly attribute effects to a single mechanism within any given stage. However, as discussed, the certification signal is strongest at stage 1—particularly for losers—when the cash award is minimal. By contrast, in stage 3, the marginal certification effect is weakest (additional publicity from the same endorser is subject to diminishing returns) while the cash prize is largest.

We acknowledge that, at the pre-incorporation stage, even a $10,000 cash prize may have non-trivial marginal value, allowing for the launch of a prototype or performing a market test. We therefore do not interpret the stage-1 effect as a pure-certification estimate, but as a certification-dominated one. The relative magnitudes are informative: if a tenfold larger stage-3 prize ($100,000) leaves long-term outcomes essentially unchanged, it is implausible that a $10,000 stage-1 prize is the operative mechanism. We return to this point in Sect. 6.

**Fig. 1:**
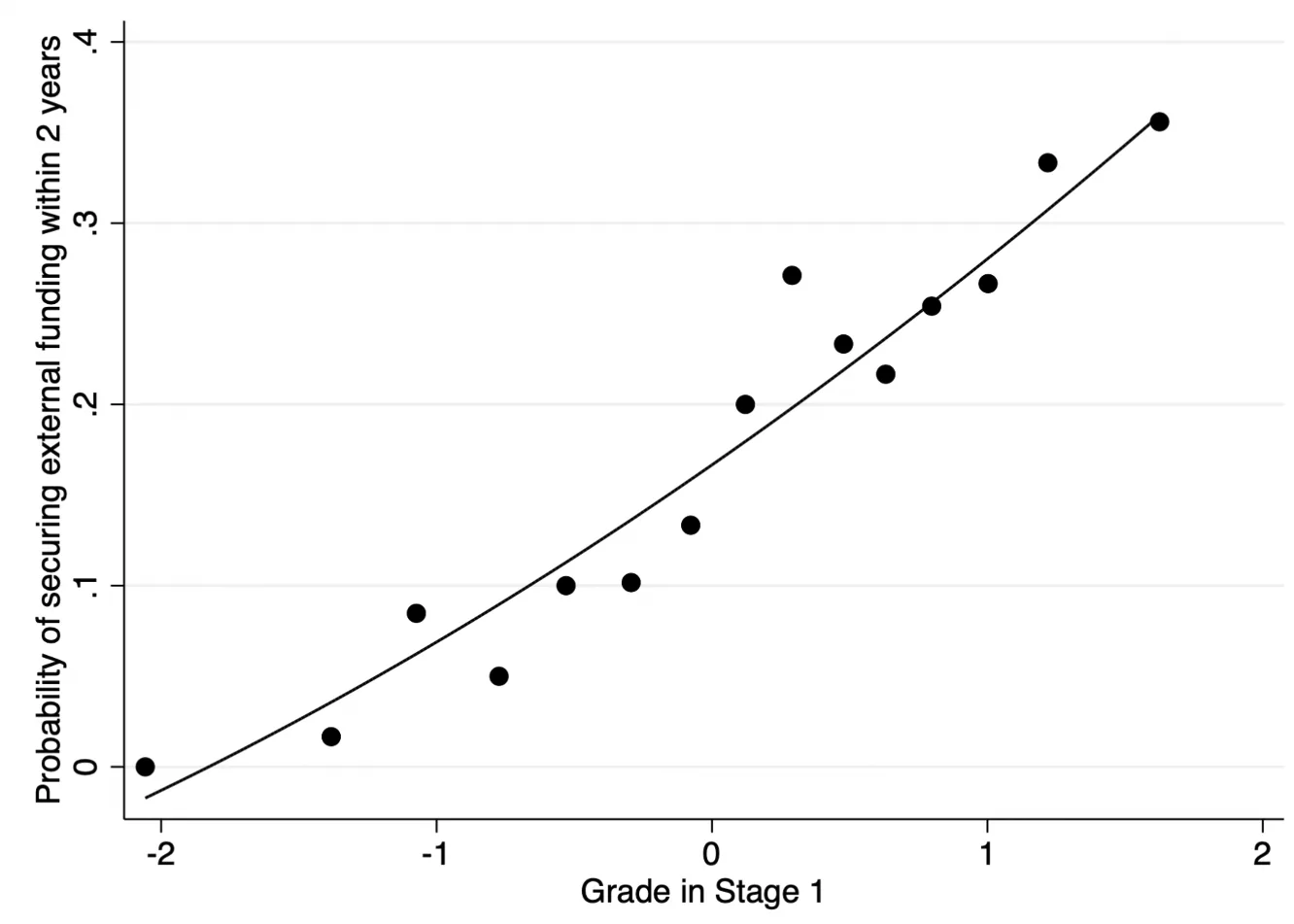
Judges are able to accurately discern startups’ true quality. *Note:* This figure shows the strong positive relationship between judges’ grades in stage 1 and the startup-specific probability of securing funding in the 2 years after the competition

It is also important to acknowledge that Venture Kick offers other value-added activities during all three stages. Indeed, the judges’ feedback and the competition in itself provide participants with opportunities to increase their network and learn how to improve their ideas or products. However, these aspects of the competition apply to all stages, making their estimation difficult. Furthermore, they are offered to both the winners and losers of each stage. Consequently, because the networking and learning opportunities lift all boats, the presence of these unobserved value-added activities does not threaten our identification strategy.

### Grades to control for startup quality

To estimate the treatment effect of winning each stage, we must deal with the fact that higher quality startups (Q) are more likely to win the competition (W) and achieve better outcomes (Y). The empirical challenge lies in disentangling the treatment effect from the “startup quality” effect. We address this challenge by using detailed data on grades. As we document below, judges’ grade (G) can be used to capture the startups’ intrinsic quality. Controlling for startup quality, we can then isolate the treatment effect of winning a stage of the competition.

Specifically, failure to observe Q when estimating the effect of winning on outcomes induces severe omitted–variable bias: we cannot separate the impact of W from that of Q. In our context, judges assess quality via a pre–award grade, potentially imperfectly G=Q+u. Judges decide who wins, so winning is perfectly predicted by G (not by Q): $W = {\textbf {1}}\{ G > c \}$, where c is the threshold.

At one (unlikely) extreme, suppose judges perfectly observe quality (G=Q). In that case, G explains outcomes for both winners and non-winners, while W captures the discrete jump in Y created by winning the competition—precisely the certification and cash effects we aim to measure. Here, correlation between G and Q affects precision (larger standard errors) rather than consistency. At the other (similarly unlikely) extreme, suppose G is random noise (G=u). Then, G is unrelated to true quality Q ($G \perp \!\!\!\perp Q$), and because W depends only on G, W is as-good-as random with respect to Q. If G has no direct effect on Y (beyond operating through W), the coefficient on W identifies the treatment effect even in this limiting case.

**Table 2:** Relationship between judges’ grades and future success

	(1)	(2)	(3)	(4)	(5)	(6)	(7)
	Survival 2	Survival 3	Survival 4	Survival 5	Survival 6	Survival 7	Survival 8
*Panel A: Stage 1*							
Standardized grade	0.198***	0.222***	0.202***	0.185***	0.144***	0.117**	0.0439
	(0.0271)	(0.0285)	(0.0307)	(0.0325)	(0.0421)	(0.0533)	(0.0662)
*Panel B: Stage 2*							
Standardized grade	0.128***	0.0943**	0.0757*	0.0688	0.0302	0.0540	0.0711
	(0.0340)	(0.0401)	(0.0431)	(0.0514)	(0.0485)	(0.0596)	(0.0775)
*Panel C: Stage 3*							
Standardized grade	0.0789**	0.0814**	0.127***	0.152**	0.134*	0.135	0.109
	(0.0339)	(0.0349)	(0.0419)	(0.0631)	(0.0773)	(0.116)	(0.132)
Year FE	Yes	Yes	Yes	Yes	Yes	Yes	Yes
Competition FE	Yes	Yes	Yes	Yes	Yes	Yes	Yes
Industry FE	Yes	Yes	Yes	Yes	Yes	Yes	Yes
University FE	Yes	Yes	Yes	Yes	Yes	Yes	Yes
Observations stage 1	657	555	457	387	297	211	119
Observations stage 2	354	305	256	214	172	123	77
Observations stage 3	179	157	135	114	95	70	48

*Note:* This presents results of linear regressions of survival status on grades received during the stages 1 through 3. The dependent variable is the survival status 2 years after the competition, *Survival 2*, in Column (1), all the way to 8 years after the competition, *Survival 8*, in Column (7). The grade variable is standardized at the stage level. The level of observation is a startup in a competition round. Errors are clustered by competition round. Asterisks *, **, and *** denote statistical significance at 10%, 5%, and 1% levels, respectively

The truth is likely to lie somewhere in the middle, with grades correlated with quality. Specifically, we argue that these grades are an informative proxy for startup quality because they strongly predict a startup’s success both in later stages and after the competition. Looking back at Table 1, we see that judges can, on average, already predict in stage 1 who the eventual winners of the competition will be. The average grade they give during the first stage to the startups that will eventually lose in stage 2 (4.22) is lower than for losers of stage 3 (4.34) and final winners (4.49). This difference cannot be due to favoritism or judges sticking with their first choice because the jury changes at each stage. More importantly, judges’ ability to predict success also applies to post-competition performances. Figure 1 shows that there is a clear positive relationship between judges’ grades in stage 1 and a startup’s probability of raising subsequent external funding in the 2 years after the competition.^20^

More formally, Table 2 displays the linear relationship between the grades given in stages 1, 2, and 3 and survival 2 to 8 years after the competition.^21^ We see that these grades are a reliable predictor of future survival rate, especially in the short term. A one-standard-deviation increase in grade in stage 1 is associated with an increase in survival rate 3 years after the competition of 23 percentage points. In the longer run, as the sample size decreases and startups evolve, this relationship understandably fades. Stage 3’s lower coefficients in the first 3 years of the competition are explained by the minimal variation in the dependent variables. Indeed, 100% of the winners of stage 3 are alive after 2 years, while 96% of them are still alive after three and 4 years. Judges show the same capability to predict success if we use funding variables as outcomes or control for winning stages of Venture Kick.

**Fig. 2:**
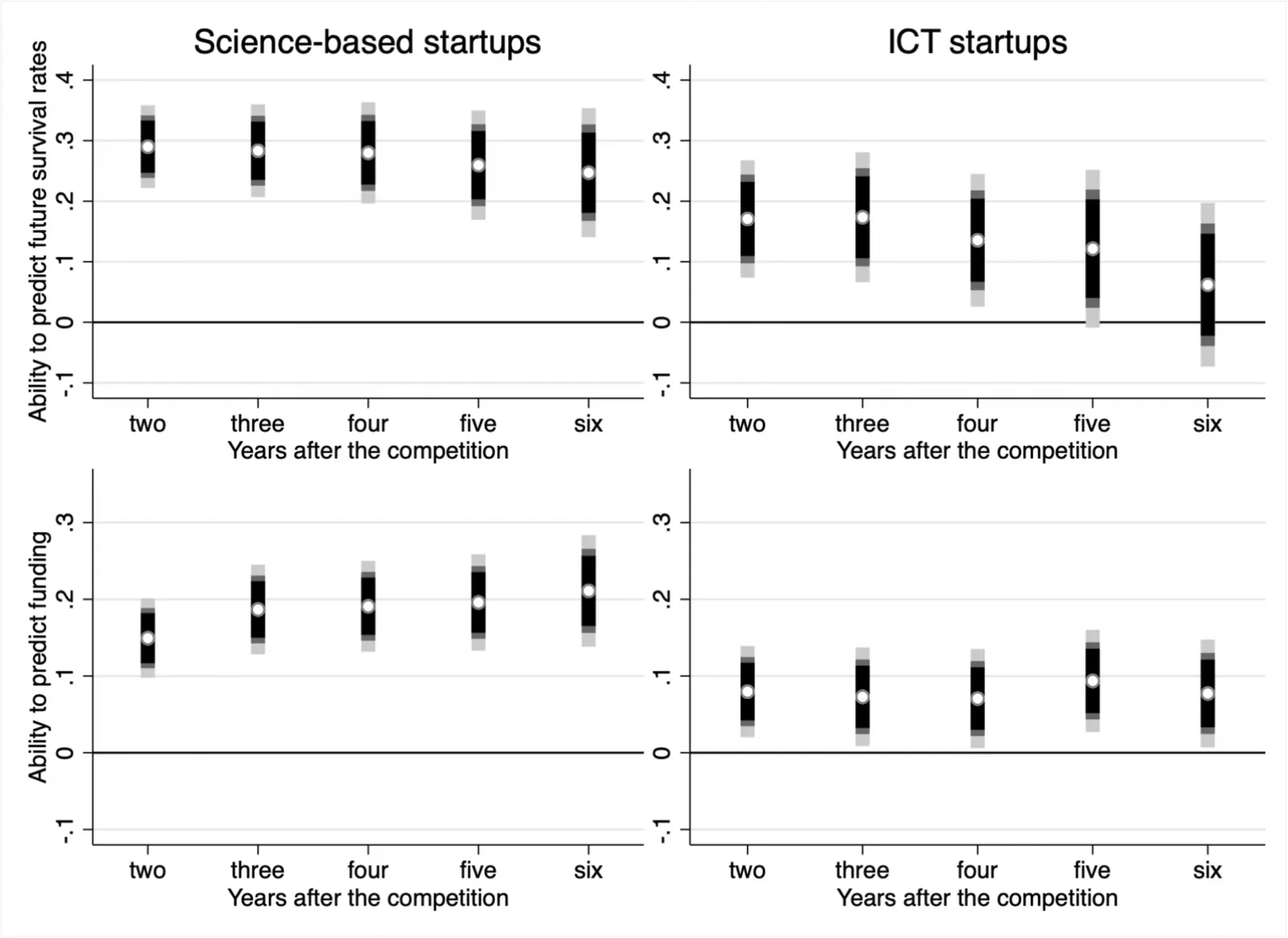
Relationship between a one-std.-deviation increase in grades and future outcomes. *Note:* The top panels display the coefficients of Table 2 (i.e., how well the judges’ grades predict survival 2 years after the competition) using interaction terms to differentiate between technology subgroups. The bottom panels show the relationship between grades and whether startups have secured financing after the competition. Errors are clustered by competition round. The confidence intervals are at the 10%, 5% and 1% levels, respectively

However, the ability of Venture Kick’s judges to objectively assess each startup’s quality varies depending on the startup’s technology, in line with previous findings in the literature (Scott et al., 2020). Our data show that Venture Kick’s experts are better at discerning between high- and low-quality early-stage science-based startups (e.g., medtech, biotech, materials and chemicals) than ICT startups. The top panel of Fig. 2 shows the coefficients of the regressions in Table 2 using interaction terms to differentiate between two technology groups (ICT startups vs. all other remaining startups, which are mostly science-based startups).^22^ In the bottom panel, the outcome variable captures whether or not a startup managed to secure funding after the competition. Overall, Venture Kick’s jury excels at evaluating the quality of science startups, and its grading remains informative in the long term. However, the grading received by an ICT startup is a weaker predictor of future business success.

We see two reasons why the Venture Kick jury is better at assessing science-based startups than ICT startups. First, the majority of the jury members are self-declared scientists. There are more than four times as many jury members with a background in science than in ICT. Second, the success of science-based startups depends on elements that are more “objective” compared to consumer products or ICT startups (e.g., hard evidence of a technological advance vs. projection of the number of users of an app). Along this line, science startups in our sample that are alive 5 years after the competition have 2.6 patents on average compared to only 0.34 for ICT startups.

This heterogeneity in the judges’ grades’ predictive power is important. Indeed, an institution’s certification of quality relies on its ability to correctly assess said quality. Because it is harder for Venture Kick’s experts to objectively evaluate ICT startups, their certification is less trustworthy than for science startups.^23^ Therefore, as we hypothesized in Section A.1, winning stage 1 should be more valuable for science ventures than for software-focused ones.

### Empirical specifications

We now have all the building blocks to estimate the certification (winning stage 1) and cash prize (winning stage 3) effects. We start by estimating these effects over the whole sample by implementing the following regression:

1 $$\begin{aligned} Y_{i,s}&= \alpha _{i,s} + \delta _1 WinsStage_{i,s} + \beta _1 StartupQuality_{i,s} \nonumber \\&\quad + \gamma _{year_s/comp/ind/uni} + \epsilon _{i,s} \end{aligned}$$

where *i* represents the startup and *s* the stage. The dependent variable $Y_{i,s}$ is either a survival or a funding outcome. The *Survival 2* to *Survival 8* variables take a value of 1 if a startup is alive 2 to 8 years after the competition and 0 otherwise. *Funding 2* to *Funding 8* are also binary variables that take a value of 1 if a startup secures non-Venture Kick funding 2 to 8 years after the competition. In some specifications, we also look at *Funding*, which does not impose any time restriction on receiving funding.^24^ Finally, *Rounds 2* to *Rounds 8* are the number of funding rounds that a startup manages to secure 2 to 8 years after the competition and take values from 0 to 10. Fixed effects for year ($\gamma _{year_s}$), competition ($\gamma _{comp}$), industry ($\gamma _{ind}$) and university ($\gamma _{uni}$) are also added. The coefficient $\delta _1$ measures the treatment effect of winning each stage, whereas $StartupQuality_{i,s}$ is proxied by the startup’s grades. Given the data structure and the fact that one firm’s win is another firm’s loss, errors are clustered by competition round. We use OLS as our main specification.^25^ In an extension, we add control variables and implement errors-in-variables regression models to account for the fact that grades might be an imperfect proxy of startup quality.

Equation 1 allows us to estimate the average treatment effect of venture competition, but it is silent on industry-specific effects. We therefore augment Eq. 1 by including interaction terms to test our predictions that venture competitions’ rewards have a different effect depending on the startups’ characteristics.

We divide the startups in our sample into two technology groups: science-based and ICT. This separation allows us to identify the impact of the competition on $high\_info$ and $low\_info$ startups. As discussed in Sect. 5.2, judges are better able to assess the quality of science-based startups ($high\_info$) than ICT startups ($low\_info$). Moreover, startups in these two groups typically have different levels of running costs. Science-based startups can require significant capital investments while ICT startups have lower costs, in part because they have recently benefited from technological shocks that have significantly reduced the cost of running a business in these sectors (e.g., the advent of cloud computing as argued by Ewens et al., 2018).

To estimate the differentiated treatment effect of each stage on ICT startups and science-based startups, we use Eq. 2 below:

2 $$\begin{aligned} Y_{i,s}^{Hetero}&= \alpha _{i,s} + \delta _1 WinsStage_{i,s} + \delta _{2}WinsStage_{i,s}*ICT_i \nonumber \\&\quad + \beta _{1}StartupQuality_{i,s}\\&\quad + \beta _{2}ICT_i + \beta _{3}StartupQuality_{i,s}*ICT_i \nonumber \\&\quad + \gamma _{year_s/comp/uni} + \epsilon _{i,s}\nonumber \end{aligned}$$

Compared to Eq. 1, we now allow for a different effect of winning each stage between sectors. The coefficient $\delta _1$ is now the effect of winning a given stage for science-based startups, and $\delta _2$ captures the difference in effect between ICT and science startups. Consequently, the effect of winning for ICT startups is the sum of $\delta _1$ and $\delta _2$. We also allow for a different relationship between judges’ grades and future outcomes between sectors ($\beta _{3}$.)

**Table 3:** Main specification: effect of winning stage 1, the *certification effect*

	Survival after the competition			Funding after comp.	
	(1)	(2)	(3)	(4)	(5)
	Survival 2	Survival 2	Survival 2	Funding	Rounds 2
*Panel A: Average effect*					
Wins stage 1	0.437***	0.373***	0.220***	0.140***	0.249***
	(0.0351)	(0.0491)	(0.0683)	(0.0454)	(0.0890)
Standardized grade			0.119***	0.0919***	0.134***
			(0.0353)	(0.0252)	(0.0463)
*Panel B: Science vs ICT*					
Wins stage 1	0.572***	0.593***	0.410***	0.217***	0.400***
	(0.0450)	(0.0570)	(0.0870)	(0.0539)	(0.108)
Wins stage 1 × ICT	−0.304***	−0.333***	−0.315**	−0.153**	−0.281**
	(0.0677)	(0.0906)	(0.123)	(0.0753)	(0.129)
ICT	0.112**	0.127**	0.142*	0.0583	0.105
	(0.0517)	(0.0598)	(0.0792)	(0.0478)	(0.0807)
Standardized grade S1			0.137***	0.136***	0.156***
			(0.0404)	(0.0294)	(0.0550)
Grade × ICT			−0.0155	−0.0805**	−0.0678
			(0.0571)	(0.0338)	(0.0604)
Year FE	No	Yes	Yes	Yes	Yes
Competition FE	No	Yes	Yes	Yes	Yes
University FE	No	Yes	Yes	Yes	Yes
Industry FE	No	Yes	Yes	Yes	Yes
Observations	671	671	671	909	893
$R^{2}$	0.196	0.397	0.413	0.327	0.321

*Note:* This presents estimates of the effect of winning stage 1 and grading on whether the startups survived or secured financing a given number of years after the competition. Panel A displays the average effect of winning stage 1 using Eq. 1, and Panel B displays the sector-specific effects using Eq. 2. Columns (1) through (3) use the survival status 2 years after the competition as the dependent variable, with varying levels of fixed effects. Column (4)’s dependent variable is whether the startup secured financing after the competition while Column (5)’s is the number of funding rounds secured within the 2 years of the competition. The level of observation is a startup in a competition round. Errors are clustered by competition round. Asterisks *, **, and *** denote statistical significance at 10%, 5%, and 1% levels, respectively

**Fig. 3:**
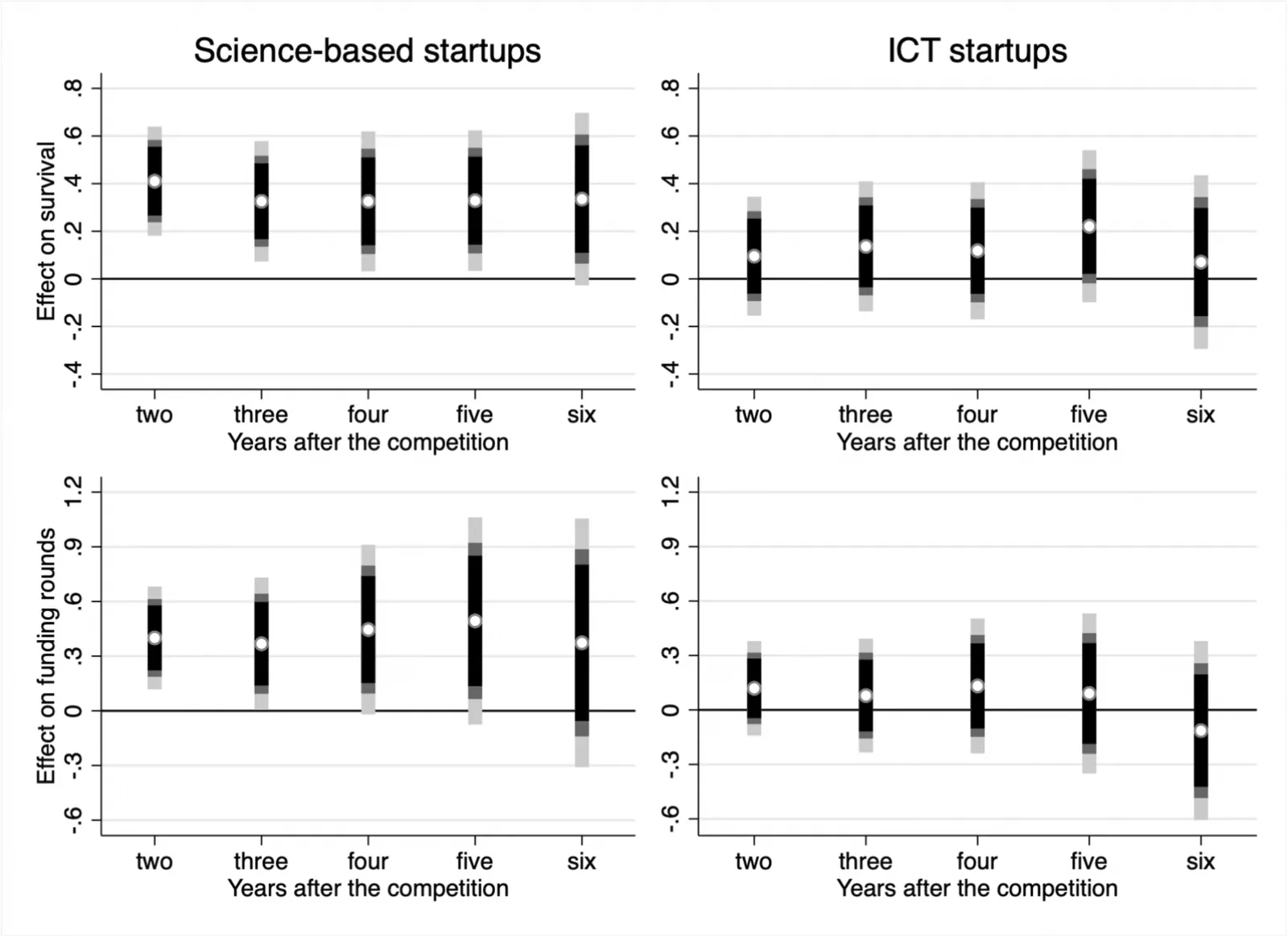
Effect of winning stage 1 on survival and funding over the years for two broad technology categories. *Note:* This presents the effect of winning stage 1 on *Survival* (top panel) and *Rounds* (bottom panel) for science-based startups and ICT startups, 2 to 6 years after the competition using Eq. 2. Errors are clustered by competition round. The confidence intervals are at the 10%, 5%, and 1% levels, respectively

## Results

### Venture Kick’s certification effect

#### Certification impacts survival and funding

We use Eqs. 1 and 2 to estimate the treatment effect of winning stage 1, our test for the certification effect, and provide the results in Table 3. Focusing, first, on the competition’s average effect in Panel A, column (1) shows that without any controls, winning stage 1 is associated with a 44-percentage-point increase in the probability of survival 2 years after the competition. This result reflects the effects of the treatment and startup quality on startup survivability. Column (2) introduces year, competition, industry, and university fixed effects. Column (3) adds the grade variable to capture the quality of the startups and is our main specification. We find that winning stage 1 increases survival after 2 years by 22 percentage points. This effect is a rather large one, given that the average survival rate of a Swiss startup 2 to 3 years after its launch is between 60 and 70% (see Appendix 6). Next columns present the effect of winning on the probability of receiving external funding after the competition (column 4) and the number of funding rounds concluded in the 2 years following the competition (column 5). Here, we look at both funding outcomes with and without the 2-year restriction to show that results do not depend on this parameter. As with survival rates, winning stage 1 has a significant positive impact. It increases the probability of securing financing after the competition by 14 percentage points and increases the number of funding rounds within 2 years by 0.25. To put these numbers into perspective, if the average startup in our sample wins stage 1, its probability of securing financing after the competition rises from 18 percentage points to 32 percentage points, a nearly 80% increase. As with survival, the treatment effect of winning stage 1 on subsequent financing is quite large.

#### The certification effect varies across technologies

Results in Panel A tell us that, on average, certification improves startups’ business success. However, these average effects conceal the fact that Venture Kick might have a heterogeneous effect on participating startups. Panel B investigates that possibility. It presents the differential effect of Venture Kick on ICT and science-based startups. Focusing on columns (3) to (5), it appears that winning stage 1 has a positive and significant impact on science-based startups’ business success. Winning stage 1 increases their survival rate 2 years after the competition by 41 percentage points. It is also associated with a rise of 0.4 in the expected number of funding rounds secured within 2 years and a jump in the probability of securing funding of 22 percentage points. However, as we predicted, the certification effect is significantly reduced for ICT startups. The difference in effect is such that ICT startups do not see any significant benefit from winning stage 1 (see Fig. 3). These results confirm that Venture Kick’s certification effect varies across sectors, in line with Proposition 1. The certification has significantly less impact on the outcome of ICT startups than it has on the outcome of science-based startups, whose quality can be more objectively assessed.

#### The certification effect is long-lasting

We now turn to studying how persistent the certification effect is over time. We predict that the certification effect should be persistent, though potentially attenuating: pre-incorporation ventures evolve—they pivot, change teams, and adjust their business models—and the original signal becomes a progressively noisier predictor of current quality. Whether and how quickly that attenuation occurs is ultimately an empirical question.

We test the prediction by estimating the treatment effect of winning Stage 1 on science-based and ICT startups 2 to 6 years after the competition, using Eq. 2. The estimates are displayed in Fig. 3, which complements the results from Table 3. They show that the certification has a long-lasting effect on science-based startups’ survivability and funding prospects. Winning stage 1 increases survival rates between 33 and 41 percentage points in the 6 years after the competition. For ICT startups, expanding the time horizon does not change the conclusion: Venture Kick’s certification does not affect survival and funding.

An alternative explanation is that the observed persistence in survival reflects winners’ reluctance to shut down. However, it seems unlikely that entrepreneurs would keep operating a loss-making venture merely because they won Stage 1 of Venture Kick—whereas losing may prompt an earlier exit. Still, winners might push the venture for a few additional months, and that extra runway could facilitate fundraising and, in turn, longer survival. If so, the effect would operate as a secondary consequence of the certification value rather than a distinct mechanism.

#### Losing entrepreneurs are most impacted by the certification

Finally, we delve deeper into the exact nature of the certification effect. Because Venture Kick’s certification impacts both the winners and the losers of each stage, we cannot know for sure whether the treatment effect of winning stage 1 comes from a positive effect on the winners or a negative effect on the losers. To study this question, we compare the predicted survival rate of the average startup in our sample with the average survival rates of young Swiss and American ventures. Appendix 6 presents the full analysis. Comparing participating and non-participating startups, it seems that winning does not significantly impact survival rates. Indeed, the predicted survival probability of the average Venture Kick startup if it wins stage 1 is very close to the average survival rate of all Swiss startups. However, the predicted survival rate of this same average startup if it loses stage 1 is much lower than the national average. While this analysis is indicative rather than definitive, the finding is consistent with the idea that losers, discouraged by negative feedback, cut their losses much quicker than they would have had they not participated in the competition.

**Table 4:** Main specification: effect of winning stage 3, the *cash prize effect*

	Survival after the competition			Funding after comp.	
	(1)	(2)	(3)	(4)	(5)
	Survival 2	Survival 2	Survival 2	Funding	Rounds 2
*Panel A: Average effect*					
Wins stage 3	0.105***	0.117**	0.0477	0.171	0.262
	(0.0311)	(0.0476)	(0.0479)	(0.139)	(0.311)
Standardized grade			0.0578	0.0733	0.137
			(0.0413)	(0.0807)	(0.166)
*Panel B: Science vs ICT*					
Wins stage 3	0.0357	0.0483	-0.0205	0.142	0.195
	(0.0250)	(0.0486)	(0.0506)	(0.163)	(0.375)
Wins stage 3 × ICT	0.198**	0.287*	0.250*	0.113	0.0533
	(0.0853)	(0.146)	(0.125)	(0.221)	(0.555)
ICT	−0.198**	−0.236*	−0.223**	−0.0203	0.325
	(0.0853)	(0.120)	(0.101)	(0.132)	(0.321)
Standardized grade S3			0.0562	0.0513	0.203
			(0.0550)	(0.0974)	(0.190)
Grade × ICT			0.0470	0.113	−0.155
			(0.0727)	(0.116)	(0.285)
Year FE	No	Yes	Yes	Yes	Yes
Competition FE	No	Yes	Yes	Yes	Yes
University FE	No	Yes	Yes	Yes	Yes
Industry FE	No	Yes	Yes	Yes	Yes
Observations	178	178	178	216	216
$R^{2}$	0.057	0.470	0.485	0.522	0.557

*Note:* This presents estimates of the effect of winning stage 3 and grading on whether the startups survived or secured financing a given number of years after the competition. Panel A displays the average effect of winning stage 3 using Eq. 1, and Panel B displays the sector-specific effects using Eq. 2. Columns (1) through (3) use the survival status 2 years after the competition as the dependent variable, with varying levels of fixed effects. Column (4)’s dependent variable is whether the startup secured financing after the competition while column (5)’s is the number of funding rounds secured within the 2 years of the competition. The level of observation is a startup in a competition round. Errors are clustered by competition round. Asterisks *, **, and *** denote statistical significance at 10%, 5%, and 1% levels, respectively

### Venture Kick’s cash prize effect

#### The cash prize increases ICT startups’ survival rates...

We now study Venture Kick’s cash prize effect by estimating the treatment effect of winning the third stage. Table 4 displays the average effect of winning stage 3 using Eq. 1 in Panel A and the technology-specific effect using Eq. 2 in Panel B. Interestingly, and in contrast to stage 1, winning stage 3 does not seem to have any effect on startups’ future business success on average. However, once again, this effect hides sector-specific differences. As column (3) in Panel B shows, winning stage 3 has no impact on the survival of the science-based startups. This result is not surprising as the $100,000 prize alone covers a science startup’s expenses for a limited period of time. However, as posited in Proposition 2, startups with lower running costs can significantly extend their runway by winning Venture Kick’s cash prize. Winning stage 3 and the related $100,000 prize increases the survival rate of ICT ventures 2 years after the competition by 23 percentage points.^26^ Finally, columns (4) and (5) tell us that the cash prize has no statistically significant effect on both science-based and ICT startups’ probability of securing funding after the competition. This result is not surprising given that investors cannot claim the cash prize for themselves and that the extra money does not fundamentally change a startup’s quality. However, we refrain from drawing definite conclusions, noting that the point estimates in Panel A are similar to those in columns (4) and (5) of Table 3.^27^

**Fig. 4:**
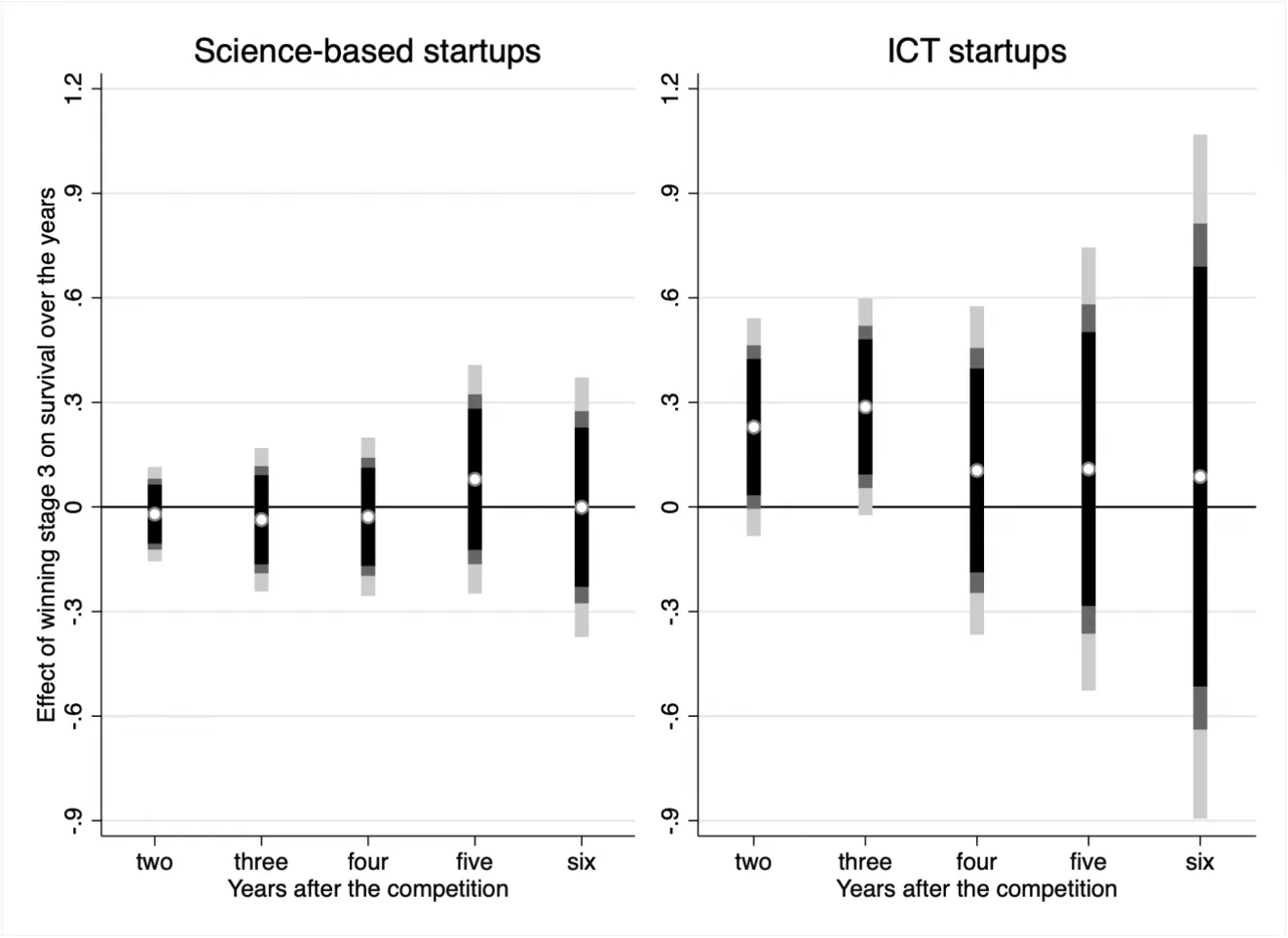
Effect of winning stage 3 on survival over the years for two broad technology categories. *Note:* This presents the effect of winning stage 3 on *Survival* for science-based startups and ICT startups, 2 to 6 years after the competition using Eq. 2. Errors are clustered by competition round. The confidence intervals are at the 10%, 5%, and 1% levels, respectively

### ... but this effect is temporary

Our second prediction about the cash prize effect is that it is short-lived (Proposition 2.a). Indeed, if the cash prize is valuable because it extends startups’ runway, then it should not have a significant impact once the prize money is exhausted. To test this prediction, we estimate the cash prize effect over the years. Figure 4 displays the results, focusing on survival (the cash prize never has any impact on funding). It confirms that the cash prize has no impact on science-based startups but, more importantly, provides an insightful visualization of the timing of the cash prize effect on ICT startups. The data suggest that winning the whole competition and the $100,000 cash prize is associated with an increase in survival rates during the first 2 to 3 years that follow the event. After that, a reasonable period for an ICT startup to consume the $100,000 prize, there is no sign that having received a cash prize has any impact on startups’ survival.

## Robustness tests

This section addresses potential concerns about our main identification strategy. We first confirm that our results are robust to including control variables and to using different versions of the grade variables. We then test the sensitivity of the results to variations in the grades’ reliability by estimating errors-in-variables regression models. Finally, we implement two alternative identification strategies that do not rely so much on the grades. Most notably, we use a regression discontinuity design (RDD) that exploits the fact that the startups ranked fourth during stage 1 receive treatment while those ranked fifth do not. In addition, we implement a propensity score matching (PSM) analysis.

### Introducing additional control variables

This section shows that our findings are robust to the inclusion of additional control variables. First, we control for motivation, which, like quality, could impact a startup’s performance both during and after Venture Kick. We proxy motivation by looking at whether startups participated in the optional kick camps offered by Venture Kick. Second, Clingingsmith and Shane (2017) have shown that founders who pitch their startups first or second in a competition are evaluated more harshly than the others. Hence, we control for the order in which the participants pitched to the jury. Third, we use another grade variable—the average of the eleven grades received—to verify that our results do not depend on the use of a PCA.

Table 10 in Appendix 7 shows that including these various controls does not change our result about stage 1’s certification effect. The variable *Motivation* is positively associated with future success, but the differential effect of winning stage 1 persists. Column (2) shows that controlling for the order of passage does not change our findings. Column (3) confirms that our results do not depend on which grade we use. We see, however, that the PCA grade in Table 3 has a slightly stronger relationship with survival than the average grade in column (3). Finally, including controls does not change the effect of stage 1 on funding outcomes.

We apply the same robustness checks to the effects of stage 3 (see Table 11 in Appendix 7). Our result remains unchanged: the cash prize only has an impact on ICT startups’ survivability.

### Allowing for quality to be imperfectly observed

As our main identification strategy relies on judges’ grading, we want to test the sensitivity of the results to changes in grades’ reliability. Indeed, even though the grades capture the best guess of nine experts about the startup quality, these grades remain an imperfect proxy of quality. Accounting for the fact that quality is observed imperfectly using errors-in-variables regression models, our conclusions remain unchanged. Table 12 in Appendix 7 shows that assuming that the grade variable has a reliability of 0.7—meaning that the noise represents 30 percent of the total variance—stage 1 continues to be the only stage with a significantly positive average effect.

Going further, the fact that judges are less adept at screening ICT startups means that grades are a relatively less accurate proxy for ICT startups’ quality than for science startups’ quality. To address this concern, we allow ICT startups’ grading to be 20 percent less reliable than science startups’. Comparing columns (2) and (3) of Table 13 in Appendix 7 shows that, if anything, assuming more imprecision in ICT grading reinforces our finding. Winning stage 1 has no effect on ICT startups, while stage 3 does not affect science-based ventures. While this approach has its limits,^28^ it shows that our results are robust to allowing for some level of imprecision in judges’ grading.

### Exploiting the sharp discontinuity in treatment

**Fig. 5:**
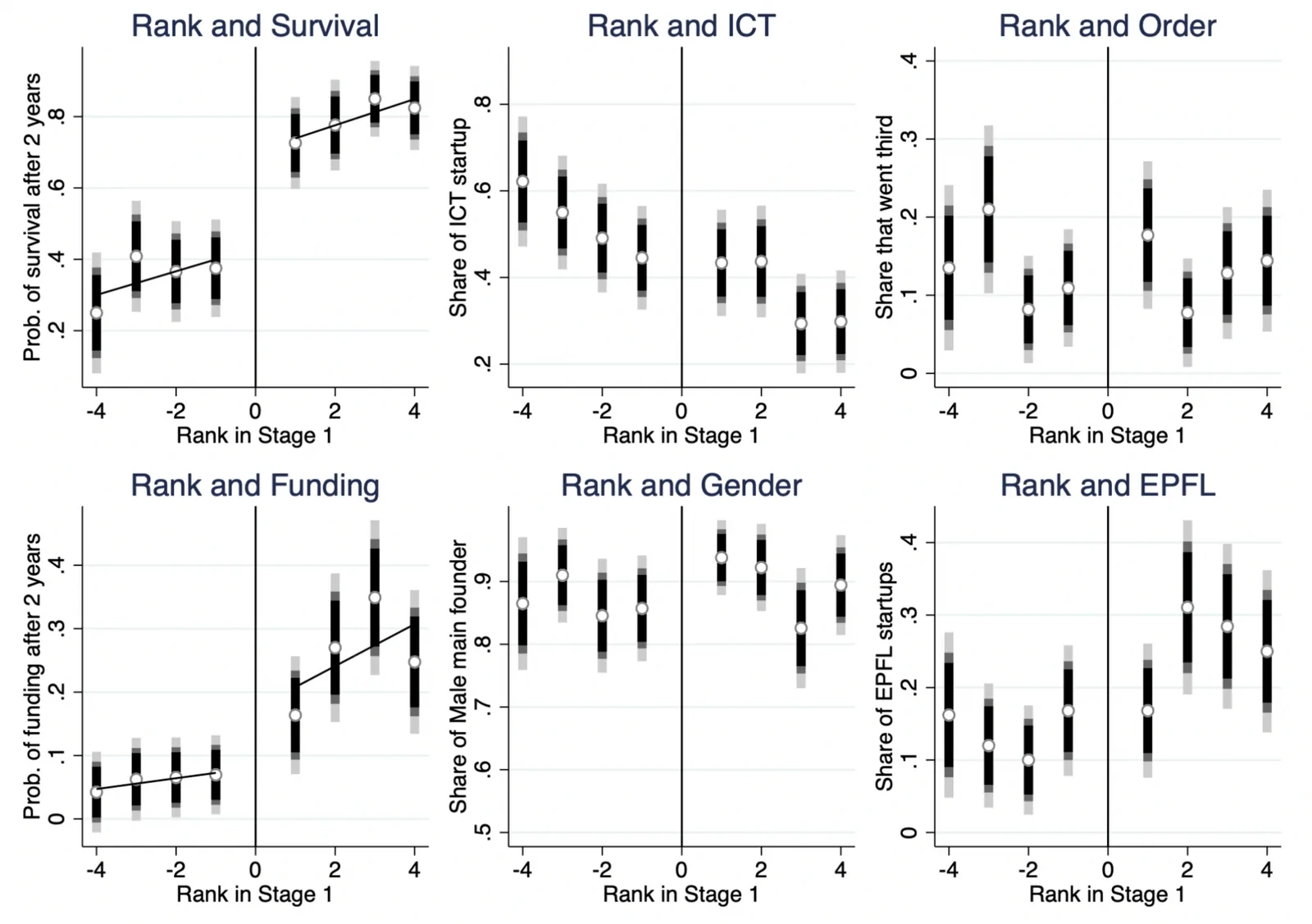
RDD: Discontinuities around the cutoff between winners and losers in stage 1. *Note:* This presents the (dis-)continuity of both our two main outcome variables as well as four selected baseline covariates around the cutoff. The values on display are the probabilities of observing startup-specific outcomes and characteristics at the time of the competition by rank in the first stage. The sample consists of all ventures in the first stage, and the result holds for all covariates. The confidence intervals are at the 10%, 5%, and 1% levels, respectively

**Fig. 8:**
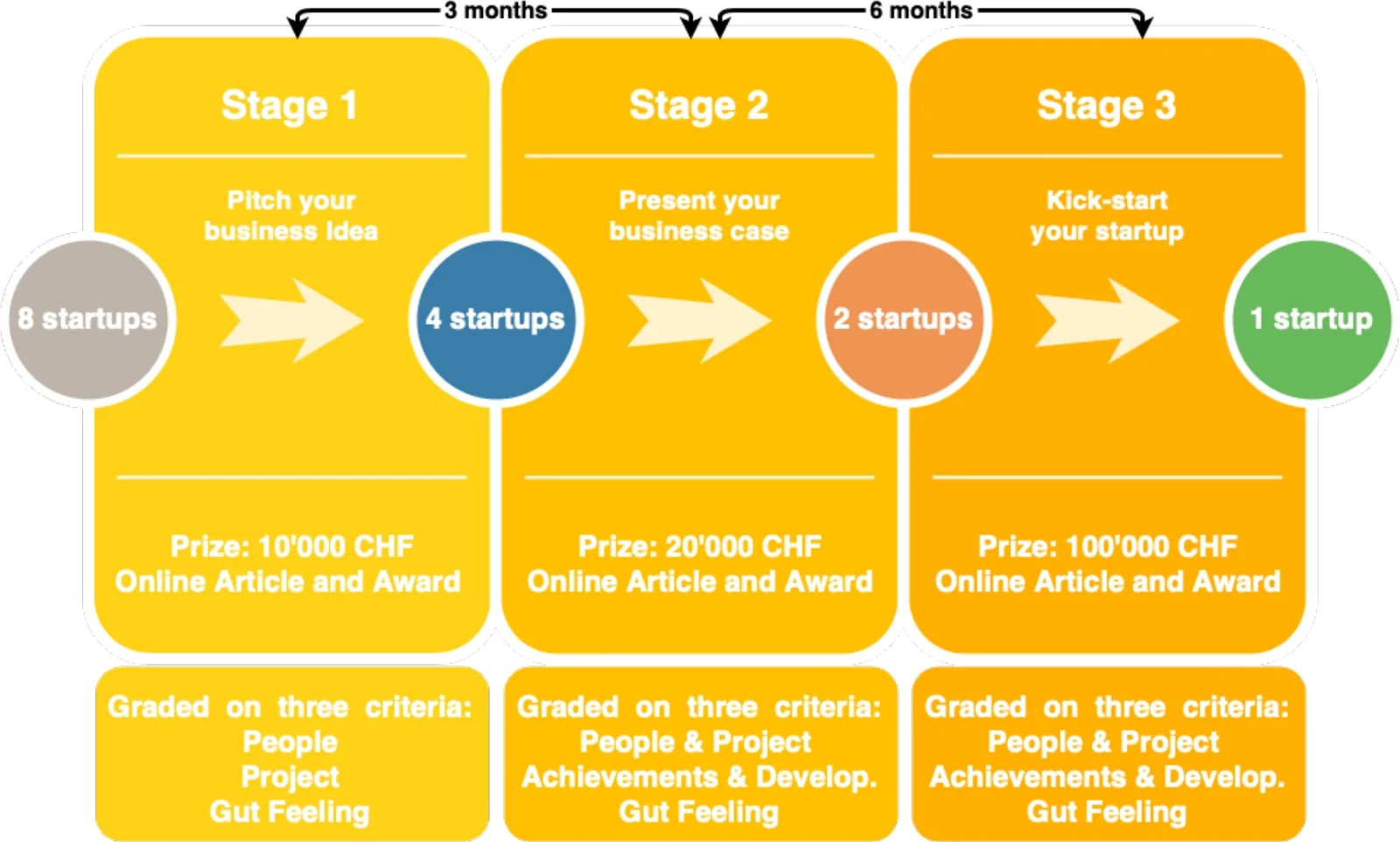
The three stages of Venture Kick. sourceEPFL

Next, we assess the robustness of our results by implementing a regression discontinuity design (RDD). We exploit the sharp discontinuity between the startups ranked fourth and fifth in the first stage, the former going on to the second stage and the latter being eliminated from the competition.^29^ We use the startup’s rank as the running variable. While having only eight startups per competition raises the issues of the discreteness of the running variable, a setting with eight competitors can still provide useful information, as shown by Howell (2017)’s study on grant competitions with an average of ten applicants.^30^ Figure 5 confirms that the use of an RDD is warranted, at least in stage 1. We can see on the top-left panel that there is an apparent discontinuity in survival rates and funding probabilities between the startups ranked fourth and fifth in stage 1. The other four panels show that there is no discontinuity around the cutoff in the probability of being an ICT startup, being the fifth startup to pitch, having a man as the principal founder, or being linked to EPFL.^31^ There is also no discontinuity at the cutoff for the other pre-competition characteristics. Our design, therefore, satisfies the RDD requirement that there should not be any discontinuity in the other baseline covariates that could cause a discontinuity in the outcome variable of interest. Finally, for an RDD to be valid, treatment should not cause rank. Specifically, the relevant identifying assumption is not that treatment is exogenous in the full sample, but that ranks are not manipulated around the cutoff (Howell, 2020; Lee & Lemieux, 2010). In our setting, this assumption is plausible: nine jury members independently grade eight ventures along eleven criteria, Venture Kick aggregates the grades into a single ranking, and the top four ventures advance as winners. Manipulating the rank-4 versus rank-5 margin would require a judge to anticipate the other judges’ scores across all criteria, which is mechanically infeasible.

Table 5 displays the results of our RDD for stage 1. Columns (1), (2), (4), and (5) are parametric RDDs using the startup’s rank in the competition as the running variable, allowing for a different slope coefficient on each side of the cutoff. Column (3) is a non-parametric RDD with a bandwidth of one rank, meaning that in stage 1, we only focus on the startups ranked fourth (best loser) and fifth (worst winner). We can see that the main results hold: stage 1 and its certification effect only have a positive and significant causal impact on science-based startups’ future performances. The size of the certification effect also remains similar. Winning stage 1 increases science-based startups’ survival 2 years after the competition by 45 percentage points and increases the probability of getting funded by 17 percentage points and the number of rounds secured by 0.45. Winning stage 1 still has no effect on ICT startups. While column (3) hints at a positive effect for ICT startups, this result is most likely a sign that with only eight possible ranks, startups are not really randomly distributed around the cutoff (fourth and fifth rank). Thus, the non-parametric RDD does not fully solve the endogeneity issue. Furthermore, we cannot implement an RDD for stage 3 because only two startups compete against each other at this stage.

**Table 5:** RDD: effect of winning stage 1

	RDD: Survival			RDD: Funding	
	(1)	(2)	(3)	(4)	(5)
	Survival 2	Survival 2	Survival 2	Rounds 2	Funding 2
Wins stage 1	0.429***	0.446***	0.394***	0.445***	0.177**
	(0.0954)	(0.119)	(0.101)	(0.138)	(0.0692)
Wins stage 1 × ICT	−0.312***	−0.347***	−0.0918	−0.384***	-0.144**
	(0.0722)	(0.101)	(0.146)	(0.130)	(0.0636)
Losers’ rank	0.0336	0.0283		0.0156	0.00867
	(0.0279)	(0.0329)		(0.0259)	(0.0148)
Winners’ rank	0.0267	0.0364		0.0374	0.0275
	(0.0221)	(0.0264)		(0.0334)	(0.0172)
Year FE	No	Yes	No	Yes	Yes
Competition FE	No	Yes	No	Yes	Yes
University FE	No	Yes	No	Yes	Yes
Industry FE	Yes	Yes	Yes	Yes	Yes
Observations	603	603	172	804	804
$R^{2}$	0.224	0.372	0.127	0.320	0.305

*Note:* This presents results of linear regressions using regression discontinuity designs (RDDs). The running variable is a startup’s rank within its competition round. A higher rank is better; in stage 1, −4 is the worst and 4 is the best. Columns (1) and (2) display parametric RDDs using the entire sample. Column (3) is a non-parametric RDD comparing startups one rank around the cutoff. Columns (4) and (5) use a parametric approach for funding. The level of observation is a startup in a competition round. Errors are clustered by competition round. Asterisks *, **, and *** denote statistical significance at 10%, 5%, and 1% levels, respectively

**Table 6:** PSM: treatment effect of winning stages 1 and 3

	Science		Internet and Software	
	(1)	(2)	(3)	(4)
	Survival 2	Rounds 2	Survival 2	Rounds 2
*Panel A: Certification*				
Wins Stage 1	0.395***	0.410***	0.131	-0.0307
	(0.132)	(0.135)	(0.185)	(0.127)
*Panel B: Cash prize*				
Wins Stage 3	0.0153	0.278	0.255***	0.407
	(0.0130)	(0.220)	(0.0730)	(0.297)
Observations stage 1	365	502	306	391
Observations stage 3	131	162	47	54

*Note:* This presents the average treatment effect from a propensity score matching for stages 1 and 3. The level of observation is a startup in a competition round. Errors are clustered by competition round. Asterisks *, **, and *** denote statistical significance at 10%, 5%, and 1% levels, respectively. We also report the differential effect for ease of interpretation. Panel A: Survival 2 differential = +0.264 (SE 0.227, *p* = 0.246); Rounds 2 differential = +0.441 (SE 0.185, *p* = 0.017). Panel B: Survival 2 differential = −0.240 (SE 0.074, *p* = 0.001); Rounds 2 differential = −0.129 (SE 0.370, *p* = 0.727)

### Matching losers and winners

**Fig. 9:**
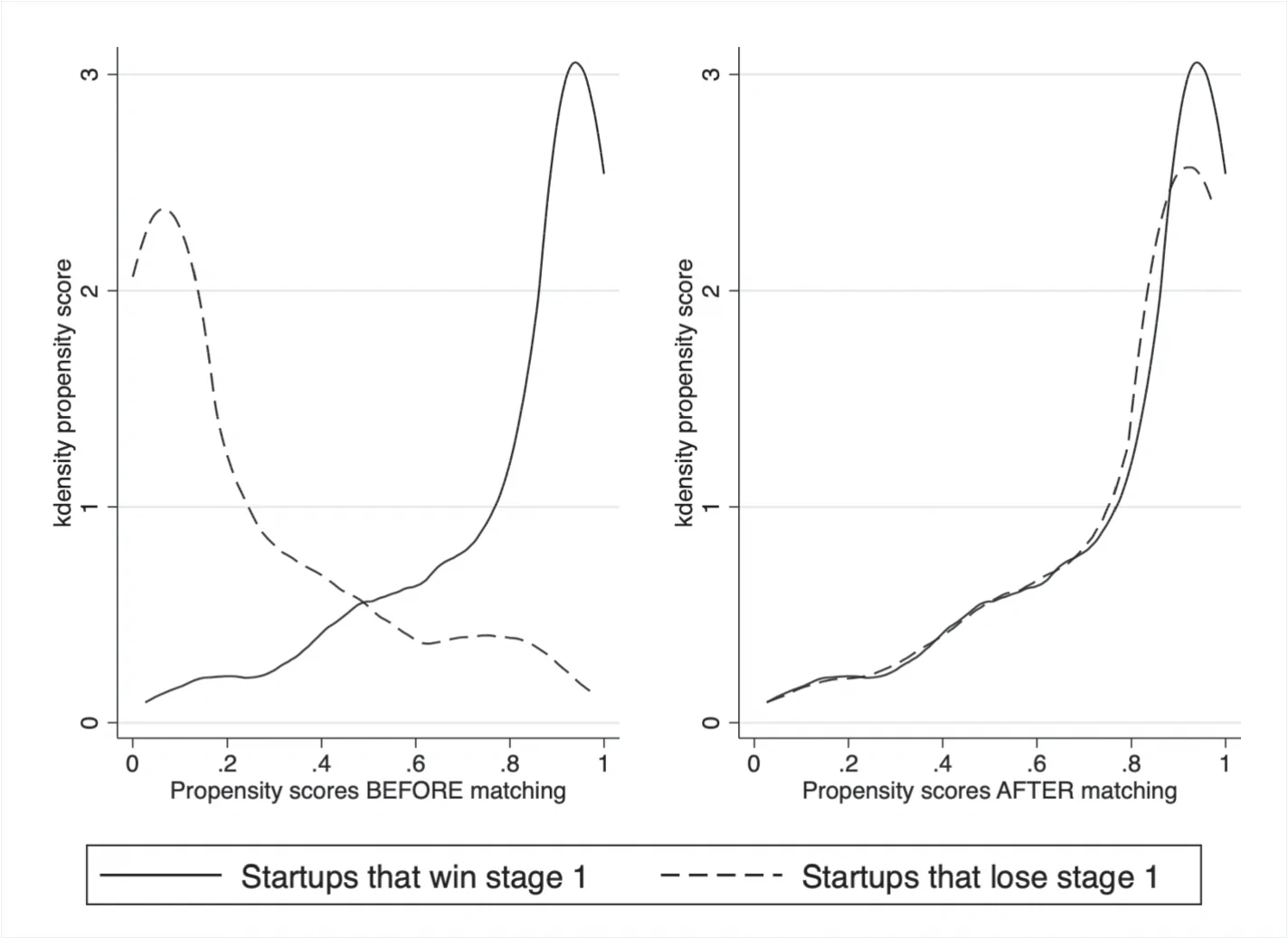
Propensity score balance across treatment and comparison groups, matching on grade, industry, university, gender, and year, stage 1

**Fig. 10:**
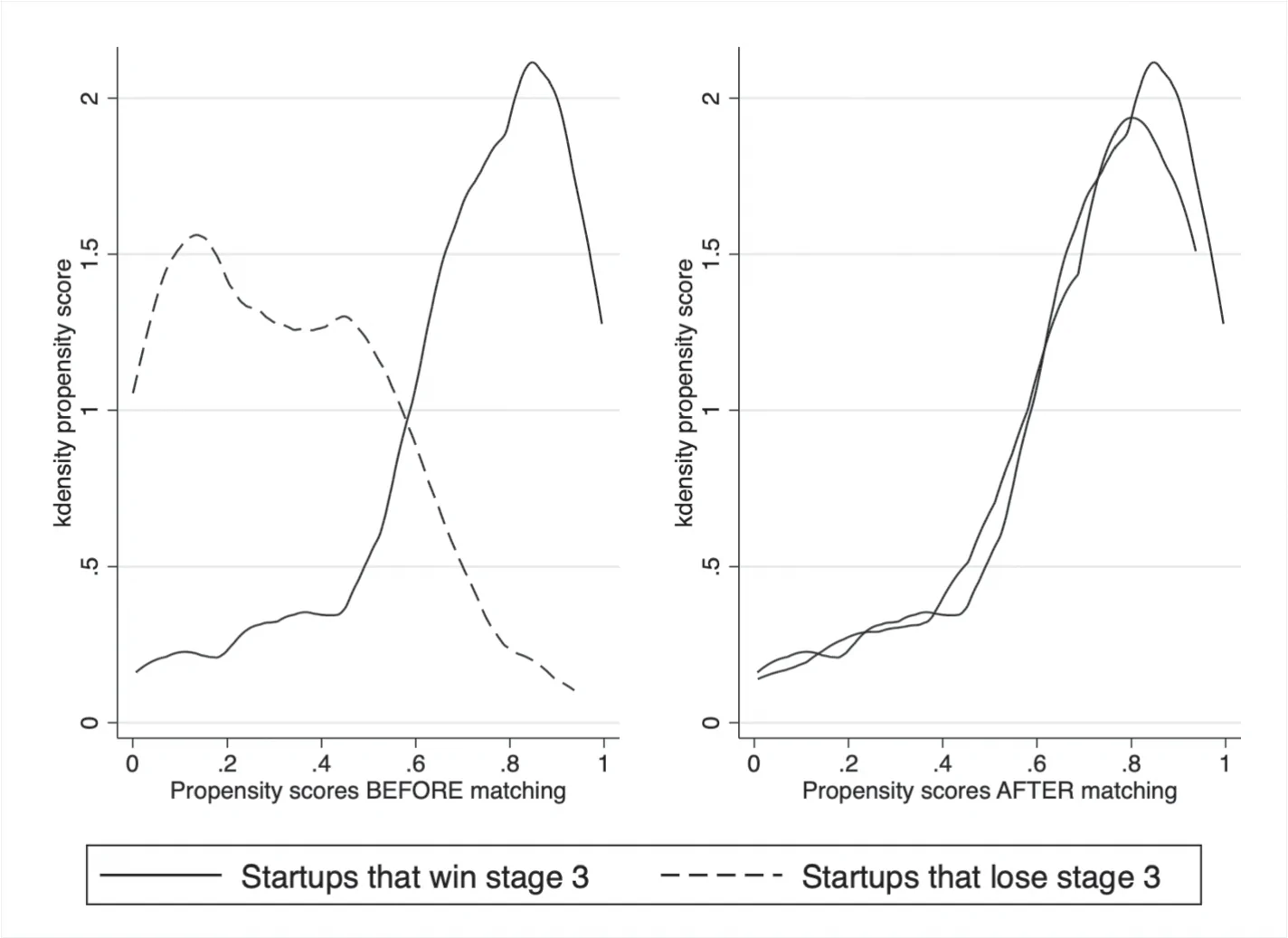
Propensity score balance across treatment and comparison groups, matching on grade, industry, university, gender, and year, stage 3

The last approach to establish the robustness of the findings involves using a propensity score matching (PSM) estimation. This technique allows us to compare similar startups whose only significant observed difference lies in whether or not they have won a given stage of Venture Kick. We match startups one to one to their nearest neighbor based on the propensity scores. We compute this score using the startups’ quality, proxied by the grade variable, their field of technology, whether they have links with EPFL or ETHZ, whether their main founder is a man, and the year of the competition. If our matching process is adequate, the matched treatment and control groups should have balanced covariates and propensity scores. Figures 9 and 10 in Appendix 8 show that the balance of scores between winning and losing startups greatly improves in the matched sample for stages 1 and 3, respectively. Table 6 shows the results of the PSM on the sample of ICT startups and the sample of science-based startups, which are in line with our main results. Moreover, the estimated treatment effects are very close to those estimated using the alternative identification strategies.

In a nutshell, we have shown that our findings are robust to alternative specifications and identification strategies. The certification effect has a significant and positive impact on the future performance of science-based startups but does not affect ICT startups. Conversely, only ICT startups benefit from the cash prize.

## Concluding remarks

The growing popularity of venture competitions has been accompanied by a rise in scholarly interest. The literature has already established that venture competitions can have a significant impact on startups’ fate. However, there is still no consensus on the exact nature of the effect of venture competitions on startup performances. Some scholars argue that venture competitions are useful because the cash prize they offer alleviates startups’ financial constraints (McKenzie, 2017; Smith & Viceisza, 2018). Others argue that what matters most is that they offer an informative certification of startup quality (Howell, 2020). In this paper, we reconcile these results and argue that, under the right circumstances, both the certification and cash prize can improve startups’ business success. We propose a theoretical model of the impact of venture competitions on participating startups. We then test the model’s predictions on a sample of 987 startups that have participated in Venture Kick, the leading Swiss venture competition. In line with previous findings, we find that venture competitions offer substantial benefits to participating startups. However, these benefits are not distributed equally among participants.

First, we find that the certification provided by Venture Kick’s first stage has a long-lasting impact on science-based startups’ survivability and ability to attract funding. It helps high-quality startups secure financing and accelerates the termination of low-quality startups.^32^ However, we find no such evidence for ICT startups. This differential effect appears to stem from differences in the reliability of judges’ ratings. As Venture Kick’s judges are less able to objectively assess the quality of ICT startups than science-based startups, their certification is less informative to entrepreneurs and investors.

Second, we find that winning Venture Kick’s third stage and its $100,000 cash prize has no effect on scien-ce-based startups but improves ICT startups’ survivabi-lity—but only in the short term. The cash prize never affects either group’s ability to attract external investors. We interpret this finding as evidence that the cash prize buys time, not quality: it extends a venture’s runway by a few months but does not fundamentally alter its prospects. The survival effect is correspondingly short-lived, concentrated in ICT ventures for whom $100,000 buys a meaningful number of operating months, and absent for science-based ventures, for whom the prize is small relative to typical capital needs. More generally, this pattern implies that we should expect cash prizes to matter most where they afford a longer runway—either because the prize is large in absolute terms or because local operating costs are low. This finding rationalizes the contrast between McKenzie (2017), who finds large cash effects in Nigeria, and Howell (2020), who finds small ones in the United States.

Furthermore, although venture competitions’ cash prize and certification have taken the limelight, venture competitions also offer other value-added activities. Indeed, venture competitions usually come with valuable mentoring and networking opportunities, which can prove crucial to the development of budding entrepreneurs (Timmons & Bygrave, 1986). Besides, tournament theory posits that competitions can be useful because they provide an incentive for participants to put some effort into improving their startup (Connelly et al., 2014). Finally, competitions can help entrepreneurs improve their startups’ ideas and products, either through the experience they gather in a learning-by-doing framework (Arrow, 1971; Minniti & Bygrave, 2001) or thanks to the judges’ feedback. We know little about the effect of these value-added activities, which present avenues for further research.

The findings presented in this paper bear implications for the design of entrepreneurial programs. The first one is that a competition’s certification can have a broad impact, both on startups’ survivability and funding prospects. Moreover, this impact can be long-lasting. However, for the certification effect to materialize, organizers need to ensure that their judges are able to accurately assess the startups’ potential. Only then will entrepreneurs and investors give credence and value to the certification. The second implication is that the cash prizes’ role might be narrower than what organizers would hope for, only extending startups’ runway without greatly impacting their long-term prospects. Organizers should, therefore, consider how much extra runway their cash prize can buy when designing venture competitions.

For entrepreneurs, the takeaway is straightforward: our results imply heterogeneous gains from participation in startup competitions. Ventures stand to benefit most when jury expertise aligns closely with their field, enhancing the credibility and reach of the certification signal. Entrepreneurs should therefore be selective, entering competitions where the panel’s expertise and the prize structure align with their needs.

## Figures and Tables

**Fig. 6:**
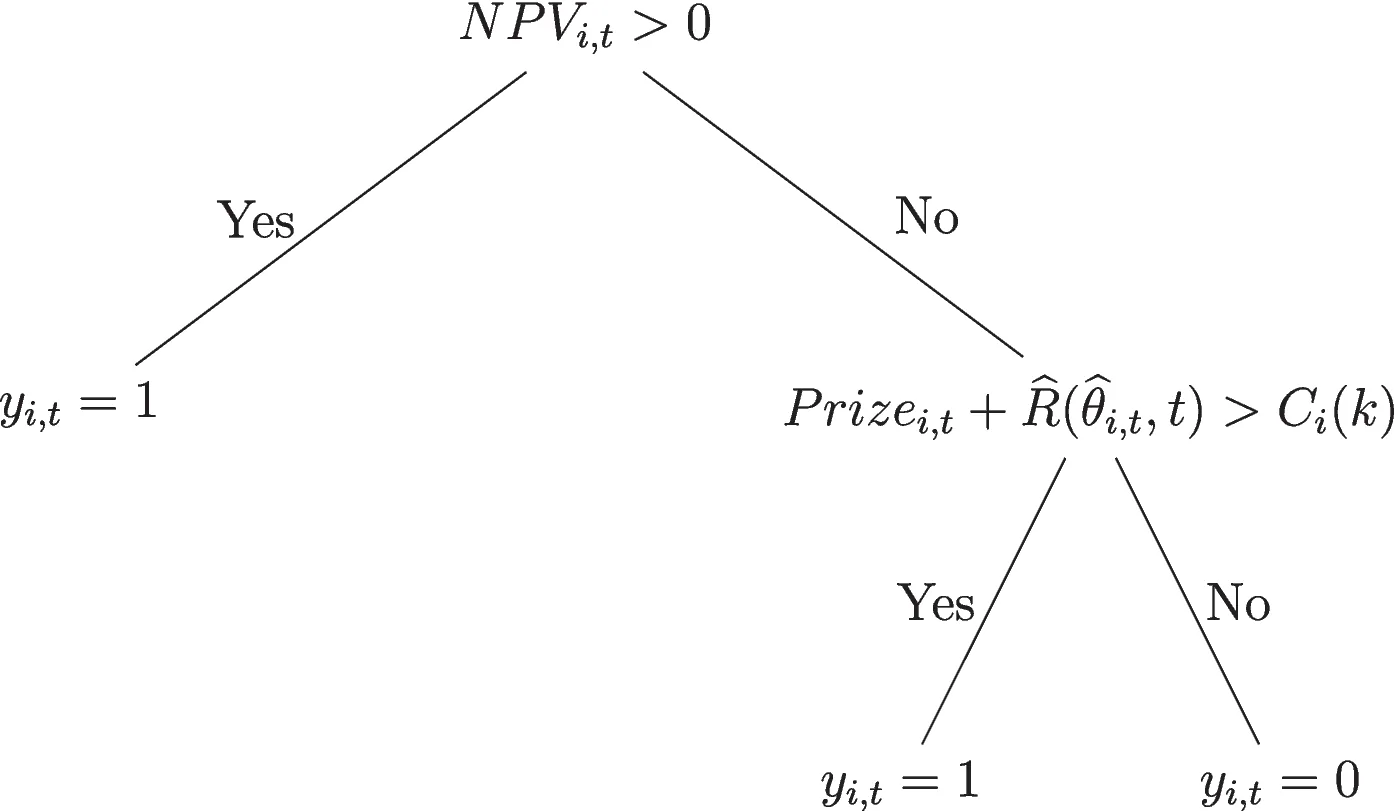
Decision tree of the entrepreneur

**Fig. 7:**
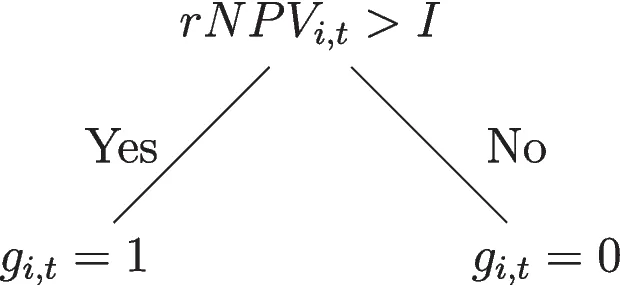
Decision tree of the investor

## Data Availability

Anonymized data are available upon request from the authors, subject to Venture Kick’s approval.

## Appendix 1. Modeling venture competitions’ effect

### A.1 The model

We develop a model of the effect of venture competitions from which we draw several testable predictions. We consider three types of agents: entrepreneurs, investors, and venture competitions. Entrepreneurs each own a startup *i* that can be one of *k* technologies assigned randomly by nature at time t=0. To operate, startups pay a running cost $C_i(k)$ each period. Entrepreneurs have to decide at the beginning of each period *t* whether to continue their activity ($y_{i,t}$) or quit. The second agents, investors, own capital and have to decide whether to invest ($g_i$) in a given startup *i*.

These decisions are taken with imperfect information because neither entrepreneurs nor investors know the true quality $\theta _i$ of startup *i*. Quality is assigned by nature at t=0 and will influence a startup’s future revenues $R(\theta _i, t)$. Revenues also depend on *t* because they typically increase as a startup transitions from the early stage to its growth stage. Entrepreneurs and investors observe imperfect signals of startup quality, which they use to predict startup quality $\widehat{\theta }(S_{i,k}, Z_{i,t})$. $Z_{i,t}$ is any information about a startup’s true quality (e.g., background of the entrepreneurial team), and $S_{i,k}$ is an external quality signal such as the one provided by venture competitions. This predicted quality is then used to estimate expected revenues, $\widehat{R}_{i,t}$. Unlike true quality $\theta _i$, which is fixed, $\widehat{\theta }_{i,t}$ evolve over time as decision-makers receive more information.

The third agents, venture competitions, provide participating startups with the following two elements. First, they offer a certification of quality $S_{i,k}$ that provides additional information to both entrepreneurs and investors, allowing them to update $\widehat{\theta }_{i,t}$. Therefore, in this model, certification serves as a type-revelation mechanism rather than as a way to address asymmetry of information. This certification can be either positive (i.e., $S_{i,k} = 1$) or negative (i.e., $S_{i,k} = -1$). Second, the competition gives a financial reward, $Prize_{i,t}$, to its winners, whose use is strictly limited to covering the costs of the startup, and which is lost if the company stops its activity.

To simplify our infinite-horizon problem formulation, we assume without loss of generality that agents are fully patient—the discount rate β is thus 1—and we set the risk-free rate at 0. The timing of the model is as follows. In period t=0, nature assigns a quality $\theta _i$ and a technology type *k* to each startup. We assume that the entrepreneurs start with a negligible amount of capital of their own. In period t=1, entrepreneurs receive information about the true quality of their startup via $Z_{i,t}$. They then adjust the estimated quality of their startup $\widehat{\theta }_{i,t}$ and, based on this estimate, decide whether to pursue the business or not. At the beginning of period t=2, some of the startups still alive are randomly selected to participate in a venture competition.^33^ Then, from period t=2 onward, entrepreneurs and investors use both $Z_{i,t}$ and $S_{i,k}$ to estimate $\widehat{\theta }_{i,t}$ and make their decisions. The majority of startups will survive in the first periods when $Z_{i,t}$ is small in absolute value. Indeed, we expect entrepreneurs to be optimistic about their probability of success when they have little information about their true quality. This overconfidence of entrepreneurs is well-documented in the literature (Koellinger et al., 2007).

#### A.1.1 The decision of the entrepreneur

The entrepreneur chooses each period whether or not to continue with their activity—if they shut down in period *t*, then $y_{i,t+j} = 0 \hspace{0.2cm} \forall \hspace{0.2cm} j \in [0, \infty ]$—based on the expected Net Present Value (NPV) of the venture at time *t*. The main determinant of the NPV is the startup’s estimated quality at time *t*, $\widehat{\theta }_{i,t}$. Accounting for the fact that a startup might fail and cease to exist, future revenues are weighted by the estimated probability of it being alive *n* periods in the future, $\widehat{y}_{i,t + n}$. Crucial to both $\widehat{\theta }_{i,t}$ and $\widehat{y}_{i,t + n}$, the information $Z_{i,t}$ is a function of its past value and a random variable $\gamma _{i,t}$ that depends on $\theta _{i}$. We allow the certification $S_i$ to have a varying impact on $\widehat{\theta }_{i,t}$ depending on how credible and trusted the certification is, which in turn is a function of startup *i*’s technology *k*. Finally, as periods pass, startups accumulate their own $Capital_{i,t}$ which is the sum of past profits (and does not encompass the non-appropriable cash prize). We assume that outside capital is available to high-quality startups to cover temporary negative cash flows (e.g., from investors). The different variables in the model are defined as follows:

$$\begin{aligned} NPV_{i,t}&= \sum _{n=0}^{\infty }\widehat{y}_{i,t + n}(\widehat{R}(\widehat{\theta }_{i,t}, t + n) - C_i(k)) \\ \widehat{\theta }_{i,t}&= f(h_k(S_{i}), Z_{i,t}) \\ \widehat{y}_{i,t + n}&= f(\widehat{\theta }_{i,t}, t + n) \\ Z_{i,t}&= Z_{i,t-1} + \gamma _{t}(\theta _{i})\\ Prize_{i,t}&= max(Prize_{i,t-1} - C_i(k), 0)\\ Capital_{i,t}&= \sum _{t=1}^{t-1}R_{i,t} - max((t-1)C_i(k) - Prize_{i,2}, 0) \end{aligned}$$

At the beginning of each period *t*, the entrepreneur can be in one of three situations depicted in the decision tree in Fig. 6. To begin with, if entrepreneur *i* estimates that their startup’s NPV is positive, they will choose $y_{i,t} = 1$. Alternatively, if an entrepreneur estimates that their project is not viable in the long term (i.e., NPV<0), they have two options. They will choose $y_{i,t} = 1$ if they expect their revenue in period *t* and what remains, if anything, of the prize to cover the cost of running the business during period *t*. Pursuing the venture one more period could allow them to increase the entrepreneur’s own capital—the prize is lost if the startup stops its activity—or receive new information, $Z_{i, t + 1}$, that could make the NPV positive. Finally, they will choose to quit their unprofitable business ($y_{i,t} = 0$) if they cannot cover the running costs at time *t*.

#### A.1.2 The decision of the investor

The investor chooses once whether to invest in a given startup ($g_{i,t} = 1$ if they decide to invest). Investing means providing *I* in exchange for a return *r* on future profits’ $NPV_{i,t}$. The *Prize* does not enter the equation because it cannot be appropriated by the investors and it does not impact the NPV. The investor’s decision is simpler than the entrepreneur’s: invest if they expect their investment to yield more than its cost and do not invest otherwise (see Fig. 7).

### A.2 Predictions drawn from our model

Before drawing some predictions from our model, we need to make explicit two assumptions on which we rely. First, we assume that the certification is an informative signal of quality, that is, $corr(S_{i,k}, \theta _i) > 0$. For the certification to be informative, judges need to be able to accurately assess startups’ quality. This first assumption is supported by the fact that, as we show in Sect. 5.2, judges’ grades in our data are highly predictive of startups’ future business success. Second, we allow the judges’ ability to identify high-quality startups to differ by technology type. This feature of our model is justified by both our data (see Fig. 2) and the literature. Indeed, Scott et al. (2020) find that experts can effectively differentiate among early-stage ventures on grounds of quality in the hardware, energy, life sciences, and medical devices sectors but cannot do so for ventures in the consumer products, consumer web and mobile, and enterprise software sectors. A possible explanation for this difference is that the success of science-based startups depends on elements that are more “objective” compared to consumer products or ICT startups (e.g., hard evidence of a technological advance vs. projection of the number of users of an app). Along this line, science startups in our sample that are alive 5 years after the competition have 2.6 patents on average compared to only 0.34 for ICT startups. Under this assumption, the competition’s certification provides more information on a life science venture than it does on an ICT venture, which implies that $corr(S_{i,k=life\_sciences}, \theta _i) > corr(S_{i,k=ICT}, \theta _i)$.

#### The certification effect

The certification, $S_{i,k}$, affects how entrepreneurs and investors estimate the startup’s probability of success, which improves their decisions (i.e., $\frac{\partial {y_{i,t}}}{\partial {S_{i,k}}}, \frac{\partial {g_{i,t}}}{\partial {S_{i,k}}} > 0$). On the one hand, startups that lose in the competition (i.e., $S_{i,k}$ goes down) see their estimated NPV going down. The fall in NPV leads entrepreneurs to close down earlier and makes it harder for them to secure funding, thus reducing wastage of resources on low-quality projects. On the other hand, winning startups now have a higher estimated NPV, which leads them to survive for longer and secure more external funding.

##### Proposition 1

*Certification is particularly useful to startups in sectors that experts are adept at screening.*

Proposition 1 stems from the fact that a certification will only be useful if it provides trusted and accurate information. However, as discussed in Section A.2, the ability of judges to screen startups varies across technology types. We define the sectors where experts can objectively identify startups’ quality as $high\_info$ and the sectors where experts struggle to do so as $low\_info$. The certification provides more information about the true quality $\theta _i$ of a $high\_info$ startup than of a $low\_info$ one. Hence, we argue that investors and entrepreneurs in the $high\_info$ sectors are more responsive to certification and significantly adjust their estimation of a startup’s quality $\widehat{\theta }_{i,t}$ and, consequently, their decisions $y_{i,t}$ and $g_{i,t}$. In other words, because $\frac{\partial {\theta _{i}}}{\partial {S_{i,k=high\_info}}} > \frac{\partial {\theta _{i}}}{\partial {S_{i,k=low\_info}}}$ we predict $\frac{\partial {y_{i,t}}}{\partial {S_{i,k=high\_info}}} > \frac{\partial {y_{i,t}}}{\partial {S_{i,k=low\_info}}}$.

##### Proposition 1.a

*The certification has a long-term impact on entrepreneurs and investors.*

Additionally, the certification effect is long-lasting. Indeed, the information provided by the certification permanently impacts how entrepreneurs and investors estimate a startup’s probability of success and, therefore, their willingness to pursue or invest in the venture.^34^

#### The cash prize effect

The cash prize temporarily covers the startups’ running costs, thereby extending their runway. Receiving a cash prize is particularly useful to startups with negative NPV, allowing entrepreneurs to pursue their activity for a few more periods than they could have otherwise. They can use this extra time to gather additional information about the startup’s true quality or to increase their capital. Arguably, cash prizes have a less direct impact on funding as investors cannot claim ownership of the prize money, and it does not increase the startup’s NPV.

##### Proposition 2

*The cash prize has a stronger impact on startups with relatively low running costs.*

We derive this proposition from the fact that cash prizes impact startups by extending their runway. As a result, the magnitude of the cash prize effect hinges on how much extra runway it provides. This, in turn, depends on the cash prize’s size relative to the startups’ running costs. Indeed, when a startup develops a capital-intensive technology, in other words, $\frac{Prize_{i,t}}{C_i(k)}$ is small, a cash prize only has a limited impact. However, the same cash prize will significantly extend the runway of startups with low running costs.

##### Proposition 2.a

*The cash prize effect is mostly temporary.*

Unlike for certification, we expect the cash prize effect to be short-lived. The cash prize allows startups of marginal quality (NPV<0) to survive for a little while. But, once the money from the cash prize runs out, entrepreneurs cannot afford to continue to run an unprofitable business and have little choice but to shut down. Therefore, the positive effect of the cash prize will be temporary for most startups. Only the small number of entrepreneurs who were able to use the extra runway to discover that their NPV was, in fact, positive will see a long-term positive effect.

## Appendix 2. Venture Kick’s grading categories in detail

**Table 7:** Grades given in each stage

	Stage 1	Stage 2	Stage 3
Grade 1	Visionary and leadership	Visionary and “Winning”	Visionary and leadership
Grade 2	“Winning” and communication	Team and complementary	“Winning” and communication
Grade 3	Credibility and expertise	Value Prop. and opportunity	Strong team and skills
Grade 4	Team and complementary	USP & IP	USP and business opportunity
Grade 5	Network and references	Business model	BP and roadmap and financing
Average	People	People and project	People and project
Grade 6	Value prop. and opportunity	Market and customers	Sold products and projects
Grade 7	Market and customers	Experts and advisory	Partnerships and alliances
Grade 8	USP and IP	Summary and investor attraction	Marketing and “Buzz”
Grade 9	Business model	Roadmap and process	Network and sources
Grade 10	Achievements and roadmap	Financial plan and strategy	Fund raised and incorporation
Average	Project	Achievements and development	Achievements and development
Grade 11	“Gut Feeling”	“Gut Feeling”	“Gut Feeling”

## Appendix 3. Venture Kick’s publicity

### C.1 Venture Kick is a respected institution

Venture Kick’s publicity is valuable because it originates from a trusted institution. These are endorsements from a member of Switzerland’s largest private science and innovation foundation and a member of Swisscom, Switzerland’s biggest telecom company:

Source: Venture Kick’s website

### C.2 The case of Imverse: a startup that won stage 1 in 2017

#### C.2.1 Venture Kick’s article

Venture Kick’s website.

#### C.2.2 Venture Kick’s tweet

Source: Twitter

#### C.2.3 Venture Kick’s logo on Imverse’s website

Imverse’s website

## Appendix 5. Principal component analysis using our eleven grades, in stage 1

**Table 8:** Eigenvalues of the components of our PCA, stage 1

Component	Eigenvalue	Difference	Proportion	Cumulative
Comp 1	8.327	7.487	0.757	0.757
Comp 2	0.841	0.397	0.076	0.834
Comp 3	0.444	0.142	0.040	0.874
Comp 4	0.301	0.046	0.027	0.901
Comp 5	0.255	0.021	0.023	0.924
Comp 6	0.235	0.060	0.021	0.946
Comp 7	0.175	0.034	0.016	0.962
Comp 8	0.141	0.029	0.013	0.974
Comp 9	0.112	0.009	0.010	0.985
Comp 10	0.103	0.036	0.009	0.994
Comp 11	0.067	0.000	0.006	1.000

**Table 9:** Component loadings for the first four components, stage 1

Grades	Comp 1	Comp 2	Comp 3	Comp 4	Unexplained
Visionary and leadership	0.298	0.402	0.329	0.066	0.078
Winning and communication	0.269	0.587	0.299	0.019	0.069
Credibility and expertise	0.311	−0.081	−0.325	0.084	0.141
Team and complementarity	0.294	0.278	−0.311	0.290	0.147
Network and references	0.303	−0.020	−0.579	0.194	0.076
Value proposition and Opp.	0.286	−0.359	0.403	0.477	0.071
Market and customers	0.316	−0.022	−0.153	−0.017	0.159
USP and IP	0.283	−0.456	0.121	−0.114	0.150
Business model	0.316	−0.269	0.219	−0.059	0.085
Achievements and roadmap	0.303	0.009	−0.061	-0.780	0.052
Gut feeling	0.334	−0.023	0.112	−0.117	0.060

## Appendix 6. Does certification impact winners or losers more strongly?

One way, albeit imperfect, of testing whether the impact of Venture Kick on survival is concentrated on losing or winning startups involves comparing the survival rates of startups in our sample with the typical survival rates of young Swiss or American ventures. The Swiss Federal Statistical Office (FSO) and the US Bureau of Labor Statistics (BLS) provide these figures. They calculated that, in the past decade, the survival rate of startups 2 years after birth has been around 67–70%, 60% after 3 years, then 54%, and finally 50% after 5 years.^35^ For Venture Kick’s participants, these numbers are respectively 61, 58, 55, and 52%. In other words, in the short term, startups that participate in the competition have lower survival rates than typical young companies, but then after 4 years, they start having slightly higher survival rates than typical startups. Additionally, we use the model in Table 3 to predict the survival rate of a startup of average quality as a function of whether it wins or loses stage 1. If this startup wins, the predicted survival rate after 2 years is 0.69, very close to the FSO value for the average young Swiss company. However, the predicted survival rate if it loses reaches 0.48, more than 22 percentage points *below* the average survival rate in Switzerland.

Taken together, these elements provide evidence that losers will cut their losses much quicker than they would have otherwise, thus avoiding costly wastage of resources. Given that this effect of Venture Kick’s certification of quality on survival rates seems to be focused on the losers, it makes sense that it is only the performance in stage 1 that causally impacts survival. Indeed, losing in stages 2 and 3 will only happen after having won stage 1 and received praise for their quality. Losing in these later stages should thus not cause entrepreneurs to drastically rethink their probability of success.

## Appendix 7. Alternative identification strategies for main effect

**Table 10:** Control variables: effect of winning stage 1

	Survival after the competition			Funding after comp.	
	(1)	(2)	(3)	(4)	(5)
	Survival 2	Survival 2	Survival 2	# Rounds	Funding
Wins stage 1	0.341***	0.399***	0.410***	0.582***	0.148***
	(0.0904)	(0.0894)	(0.0889)	(0.184)	(0.0553)
Wins stage 1 × ICT	−0.310**	−0.311**	−0.311**	−0.582***	−0.153**
	(0.123)	(0.125)	(0.127)	(0.219)	(0.0716)
Standardized grade	0.122***	0.143***		0.403***	0.134***
	(0.0405)	(0.0409)		(0.0937)	(0.0292)
Grade × ICT	0.00218	−0.0149		−0.139	−0.0692**
	(0.0569)	(0.0587)		(0.118)	(0.0331)
Motivation	0.202***				0.170***
	(0.0482)				(0.0486)
Average grade			0.133***		
			(0.0413)		
Grade × ICT			−0.0201		
			(0.0594)		
Order FE	No	Yes	No	No	Yes
Year FE	Yes	Yes	Yes	Yes	Yes
Competition FE	Yes	Yes	Yes	Yes	Yes
University FE	Yes	Yes	Yes	Yes	Yes
Industry FE	Yes	Yes	Yes	Yes	Yes
Observations	671	671	671	909	909
$R^{2}$	0.396	0.392	0.377	0.320	0.355

*Note:* This presents estimates of the effect of winning stage 1 using Eq. 2 with additional controls. Columns (1) to (3) focus on the survival outcome. Column (1) controls for motivation, Column (2) for the order of passage during the competition, and columns (3) uses an alternative grade variable. Columns (4) and (5) introduce a new dependent variable, the number of funding rounds secured, and test our control variables on *Funding*. The level of observation is a startup in a competition round. Errors are clustered by competition round. Asterisks *, **, and *** denote statistical significance at 10%, 5%, and 1% levels, respectively

**Table 11:** Control variables: effect of winning stage 3

	Survival after the competition			Funding after comp.	
	(1)	(2)	(3)	(4)	(5)
	Survival 2	Survival 2	Survival 2	# Rounds	Funding
Wins stage 3	−0.0127	−0.0135	−0.0310	0.233	0.143
	(0.0517)	(0.0483)	(0.0533)	(0.771)	(0.165)
Wins stage 3 × ICT	0.253*	0.210*	0.239*	0.317	0.113
	(0.127)	(0.119)	(0.126)	(1.149)	(0.240)
Standardized grade	0.0572	0.0580		0.427	0.0497
	(0.0552)	(0.0544)		(0.372)	(0.102)
Grade × ICT	0.0467	0.0485		−0.0362	0.116
	(0.0706)	(0.0733)		(0.531)	(0.118)
Motivation	−0.0337				−0.00756
	(0.0851)				(0.0935)
Average grade			0.0622		
			(0.0561)		
Grade × ICT			0.0501		
			(0.0769)		
Order FE	No	Yes	No	No	Yes
Year FE	Yes	Yes	Yes	Yes	Yes
Competition FE	Yes	Yes	Yes	Yes	Yes
University FE	Yes	Yes	Yes	Yes	Yes
Industry FE	Yes	Yes	Yes	Yes	Yes
Observations	178	178	178	216	216
$R^{2}$	0.468	0.475	0.472	0.457	0.518

*Note:* This presents estimates of the effect of winning stage 3 using Eq. (2) with additional controls. Columns (1) to (3) focus on the survival outcome. Column (1) controls for motivation, Column (2) for the order of passage during the competition, and column (3) uses an alternative grade variable. Columns (4) and (5) introduce a new dependent variable, the number of funding rounds secured, and test our control variables on *Funding*. The level of observation is a startup in a competition round. Errors are clustered by competition round. Asterisks *, **, and *** denote statistical significance at 10%, 5%, and 1% levels, respectively

**Table 12:** Errors-in-variables regression: effect of winning stages 1, 2, and 3

	Baseline		EiV
	(1)	(2)	(3)
	Survival 2	Survival 2	Survival 2
*Stage 1*			
Wins stage 1	0.220***	0.354***	0.257***
	(0.0683)	(0.0456)	(0.0811)
Standardized grade	0.119***	0.0610***	0.132***
	(0.0353)	(0.0222)	(0.0511)
*Stage 2*			
Wins stage 2	−0.0541	0.101**	0.0608
	(0.0666)	(0.0467)	(0.0574)
Standardized grade	0.148***	0.0427*	0.0768*
	(0.0429)	(0.0224)	(0.0407)
*Stage 3*			
Wins stage 3	0.0477	0.0782**	0.0569
	(0.0479)	(0.0390)	(0.0371)
Standardized grade	0.0578	0.0226	0.0407
	(0.0413)	(0.0190)	(0.0298)
Year FE	Yes	No	No
Competition FE	Yes	No	No
Industry FE	Yes	No	No
University FE	Yes	No	No
Observations stage 1	671	671	671
Observations stage 2	353	353	353
Observations stage 3	178	178	178

*Note:* This presents results of standard as well as errors-in-variables regressions. Column (1) reports our baseline specification, and column (2) is a standard regression without fixed effects which serves as a benchmark for column (3), which shows our errors-in-variables regression with our grade variable having a reliability of 0.7. The level of observation is a startup in a competition round. Errors are clustered by competition round. Asterisks *, **, and *** denote statistical significance at 10%, 5%, and 1% levels, respectively

**Table 13:** Effect of winning stages 1 and 3 using an errors-in-variables regression

	Baseline		EiV
	(1)	(2)	(3)
	Survival 2	Survival 2	Survival 2
*Stage 1*			
Wins stage 1	0.410***	0.499***	0.512***
	(0.0870)	(0.0601)	(0.0959)
Wins stage 1 × ICT	−0.315**	−0.323***	−0.354*
	(0.123)	(0.0905)	(0.210)
Standardized grade	0.137***	0.0558*	0.0459
	(0.0404)	(0.0295)	(0.0617)
Grade × ICT	−0.0155	0.0107	0.0333
	(0.0571)	(0.0445)	(0.143)
*Stage 3*			
Wins stage 3	−0.0205	0.00316	0.00697
	(0.0506)	(0.0479)	(0.0271)
Wins stage 3 × ICT	0.250*	0.207**	0.200**
	(0.125)	(0.0806)	(0.0793)
Standardized grade	0.0562	0.0244	0.0216
	(0.0550)	(0.0236)	(0.0282)
Grade × ICT	0.0470	0.0125	0.0199
	(0.0727)	(0.0380)	(0.0648)
Year FE	Yes	No	No
Competition FE	Yes	No	No
University FE	Yes	No	No
Industry FE	Yes	Yes	Yes
Observations stage 1	671	671	671
Observations stage 3	178	178	178

*Note:* This presents results of standard as well as errors-in-variables regressions. The top panel shows the effect of winning stage 1, and the bottom panel shows the effect of winning stage 3. Column (1) reports our baseline specification. Column (2), a standard regression without fixed effects, serves as a benchmark for column (3), that shows our errors-in-variables regression where the ICT grade interaction has a reliability of 0.8. The grades attributed to science startups are assumed to be reliable. The level of observation is a startup in a competition round. Errors are clustered by competition round in column (1). Asterisks *, **, and *** denote statistical significance at 10%, 5%, and 1% levels, respectively

## Footnotes

1 One event that is credited with having sharply reduced the cost of starting a business is the introduction of Amazon’s Web Services (AWS) in early 2006, a cloud computing service (Ewens et al., 2018).

2 Other aspects of venture competitions such as mentoring and networking or the opportunity to learn can benefit participating startups. However, our data are not suited to studying these value-added activities as all participants enjoy them. Hence, we focus on the cash prize and certification effect.

3 Fifty-seven percent of the Shark Tank’s contestants receive funding of an average amount of around $145,000.

4 Dealroom.co, a global provider of data and intelligence on startups and tech ecosystems, estimates that Switzerland had close to 280 million venture capital dollars invested per one million inhabitants in 2024.

5 This ranking is based on CrunchBase’s “CB Rank (Company).”

6 In 2019, Venture Kick increased the money given in the second stage from $20,000 to $40,000, increasing the total amount received by the final winner from $130,000 to $150,000. The conditions attached to the funding have also changed. The initial $10,000 is still given as a grant, but the remaining $140,000 is now a convertible loan with an interest rate of 5%. As these changes occurred outside of the time frame of our study, we do not look into their implications.

7 The organizers do not systematically track reasons for rejection. That said, most rejections stem from failure to meet the participation criteria—typically a lack of affiliation with a tertiary-education institution or the venture being already incorporated or funded. Less commonly, applications are declined because the venture appears low quality, the registration package is weak, or the submission shows limited effort.

8 Unfortunately, we cannot link the expertise of the judges to their choice of winning startups as jury participation is anonymized.

9 Table 7 in Appendix 2 shows the exact grading categories given during the three stages.

10 Appendix 3 provides concrete examples of this publicity.

11 One thing that we do not know is the startups’ founding date, and, therefore, cannot include the startups’ age in our estimations. However, the participation requirements (i.e., no professional investors already involved and no incorporation) ensure that the startups are at the same stage of development when starting the competition.

12 The first selection of 6000 applications is made by an algorithm after a rapid assessment (10 min approximately). In the second stage, the remaining 4510 business plan applications are marked by an evaluator drawn from a team of 20 individuals, with a typical plan taking 30-45 min to mark.

13 We use this approach because we have noticed that the grade variable it produces for each stage is a better predictor of future startup success than any other aggregation method (e.g., average of the grades).

14 The survival status of the startups that competed in 2016 and 2017 was deemed “too young to tell” because most of them had less than 2 years of existence at the time of the data gathering process (i.e., spring to summer 2018).

15 The Internet Archive is a San Francisco based non-profit digital library and its WayBack Machine archives the entire web: WayBack Machine.

16 Unfortunately, as we show in Table 1, exit is a very rare event, happening to only 3% of the startups in our sample. This lack of variability prevents us from using exit data in our regression analyses.

17 These data were collected in early 2020 and, therefore, contain two more years than the survival variable.

18 To view some endorsement and details of Venture Kick’s publicity, see Appendix 3.

19 While we do not show any estimate of stage 2’s treatment effect in this paper, we find that Venture Kick’s second stage does not have any impact on startups’ future business success. One potential reason behind this result could be that stage 2 simply lacks any distinctive reward that might significantly affect a startup’s performance.

20 Note that because grades are never made public, investors cannot condition on grades when deciding on funding.

21 We do not control for winning the stage at this point because we want to focus on the grades. Moreover, the relationship between grades and future performance remains the same whether we control for it or not.

22 The startups classified as science-based fall in the following categories: medtech, electronics and mechanics, biotech, cleantech, materials and chemicals, micro-nano technology, and others. ICT startups are those in the internet and mobile and software categories.

23 In additional analyses (available upon request), we show that the average standard deviation of judges’ grades is consistently higher for ICT than science-based ventures—a clear signal of greater disagreement between judges.

24 We use both the time-restricted and unrestricted *Funding* variable because both have their own appeal. Focusing only on the first 2 years, for example, prevents our funding outcome from being too heavily influenced by the startups’ survival rates, as even the weakest startups will be alive and able to search for financing for a good part of these 2 years. At the same time, focusing on all the available data puts startups whose treatment effect might take longer to materialize on a more equal footing.

25 We have also estimated logit models and obtained similar findings. We report OLS regression results because they are easier to interpret. Furthermore, OLS does as well as logit, if not better, in estimating treatment effects of treatment (Angrist, 2001).

26 This ICT-specific effect is not enough to drive a positive average effect because, while ICT startups represent 43% of our sample, they only represent 17% of stage 3 winners. This result therefore cautions against using average treatment effects when analyzing the impact of venture competitions such as Venture Kick. Indeed, in our case, they hide the fact that cash prizes are actually useful for ICT startups, a growing share of the startup universe.

27 We acknowledge that the stage-3 estimates are based on a smaller sample (178 observations) than the stage-1 estimates and that the two finalists are not randomly drawn but are the top-ranked ventures of an already twice-screened pool. We therefore read the stage-3 coefficients as suggestive rather than definitive and place primary weight on the convergence across specifications, as shown further below (grade-controlled OLS, propensity-score matching, and errors-in-variables).

28 We are forced, for example, to drop most of our fixed effects due to the limited number of observations in our sample.

29 Note that because the ranks around the cutoff are equally populated by construction, there is no need to show a density test à la McCrary (2008).

30 Unfortunately, we cannot implement the RDD in stage 3 due to the reduced number of participants on each side of the cutoff. This is the reason why the RDD is not our main identification strategy.

31 EPFL is one of the two largest research-intensive technical universities in Switzerland.

32 Because the unconditional probability of winning Venture Kick is relatively high (1/8), the competition primarily functions to screen out lower-quality startups. By contrast, competitions with much lower win rates (on the order of 1–2%) serve a different purpose: identifying exceptional ventures. One might therefore speculate that the former design matters most for losing entrepreneurs—by encouraging earlier exit and limiting losses—whereas the latter design is particularly informative for outside investors, by signaling exceptionally high quality.
